# Hierarchically Porous Structured Adsorbents with Ultrahigh Metal–Organic Framework Loading for CO_2_ Capture

**DOI:** 10.1021/acsami.4c10730

**Published:** 2024-09-16

**Authors:** Solomon K. Gebremariam, Anish Mathai Varghese, Sebastian Ehrling, Yasser Al Wahedi, Ahmed AlHajaj, Ludovic F. Dumée, Georgios N. Karanikolos

**Affiliations:** † Department of Chemical and Petroleum Engineering, Khalifa University, P.O. Box 127788, Abu Dhabi 127788, United Arab Emirates; ‡ Center for Catalysis and Separation (CeCaS), Khalifa University, P.O. Box 127788, Abu Dhabi 127788, United Arab Emirates; § 3P Instruments GmbH & Co. KG, Bitterfelder Str. 1-5, Leipzig 04129, Germany; ∥ Abu Dhabi Maritime Academy, P.O. Box 54477, Abu Dhabi 127788, United Arab Emirates; ⊥ Research and Innovation Center on CO_2_ and H_2_ (RICH), Khalifa University, P.O. Box 127788, Abu Dhabi 127788, United Arab Emirates; # Research and Innovation Center on 2D nanomaterials (RIC-2D), Khalifa University, Arzanah precinct, Sas Al Nakhl, P.O. Box 127788, Abu Dhabi, 127788, United Arab Emirates; ∇ Department of Chemical Engineering, University of Patras, Patras, 26504, Greece; ○ Institute of Chemical Engineering Sciences, Foundation for Research and Technology-Hellas (FORTH/ICE-HT), Patras, 26504, Greece

**Keywords:** metal−organic frameworks, structured adsorbents, CO_2_ capture, greenhouse gases, adsorption, MOF composites

## Abstract

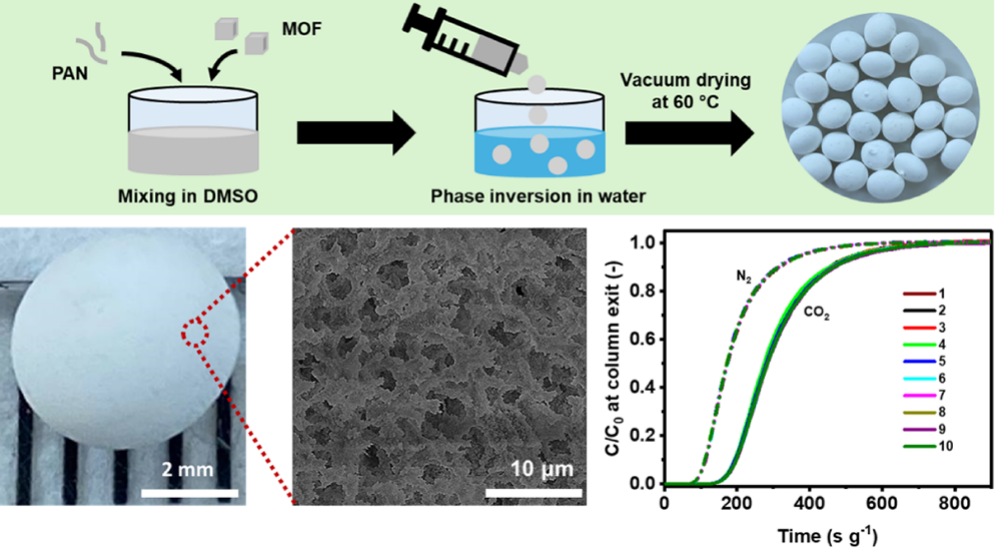

Metal–organic frameworks (MOFs) have emerged as promising candidates for CO_2_ adsorption due to their ultrahigh-specific surface area and highly tunable pore-surface properties. However, their large-scale application is hindered by processing issues associated with their microcrystalline powder nature, such as dustiness, pressure drop, and poor mass transfer within packed beds. To address these challenges, shaping/structuring micron-sized polycrystalline MOF powders into millimeter-sized structured forms while preserving porosity and functionality represents an effective yet challenging approach. In this study, a facile and versatile strategy was employed to integrate moisture-stable and scalable microcrystalline MOFs (UiO-66 and ZIF-8) into a poly(acrylonitrile) matrix to fabricate readily processable, millimeter-sized hierarchically porous structured adsorbents with ultrahigh MOF loadings (∼90 wt %) for direct industrial carbon capture applications. These structured composite beads retained the physicochemical properties and separation performance of the pristine MOF crystal particles. Structured UiO-66 and ZIF-8 exhibited high specific surface areas of 1130 m^2^ g^–1^ and 1431 m^2^ g^–1^, respectively. The structured UiO-66 achieved a CO_2_ adsorption capacity of 2.0 mmol g^–1^ at 1 bar and a dynamic CO_2_/N_2_ selectivity of 17 for a CO_2_/N_2_ gas mixture with a 15/85 volume ratio at 25 °C. Furthermore, the structured adsorbents exhibited excellent cyclability in static and dynamic CO_2_ adsorption studies, making them promising candidates for practical application.

## 1 Introduction

CO_2_ capture from a wide variety of emission sources and directly from air is of major importance for mitigating the impact of the rising global CO_2_ levels.^1^ Liquid amine-based sorbents currently represent the state-of-the-art CO_2_ capture technology on an industrial scale, yet they often suffer from drawbacks such as equipment corrosion, amine loss and degradation, and a high energy penalty associated with solvent regeneration.^2^ Adsorption technology using porous solid adsorbents has been proposed as a promising alternative to address these challenges.^3^

Metal–organic frameworks (MOFs) are promising candidates for CO_2_ adsorption due to their favorable features, including ultrahigh specific surface area and highly tunable pore surface properties.^4,5^ However, for practical applications under industrial settings, several parameters beyond CO_2_ adsorption capacity and selectivity are required,^6,7^ such as recyclability, processability, and long-term stability in the presence of moisture, acidic gases (NO_2_, SO_2_), and heat. To date, no adsorbent that simultaneously meets all the required criteria has yet been reported.^8^ For instance, the exposure of most MOFs to moisture, typically present in CO_2_-containing gas mixtures such as atmospheric air and flue gases from cement and coal-fired power plants, can lead to the decomposition or loss of crystallinity due to the relatively weak metal–ligand bonds within the framework.^9,10^ Water molecules cluster around and interact with the metal centers of MOFs, promoting irreversible hydrolysis of their metal–ligand bonds, leading to distortion or destruction of the crystal lattice.^10^ For example, Mg-MOF-74 (Mg/DOBDC) lost 99% of its surface area after 1 day of exposure to 90% relative humidity^11^ and HKUST-1 showed a 50% reduction in surface area after moisture adsorption followed by regeneration.^12^ MOF-5 decomposed into a nonporous solid under humid air exposure,^13^ while SIFSIX-3-Zn and SIFSIX-3-Cu underwent phase transformations under moisture exposure.^14^ Therefore, to mitigate operational costs associated with frequent adsorbent replacements due to moisture degradation, highly moisture-stable MOFs are essential for practical CO_2_ capture applications.

ZIF-8, characterized by its zeolite-like three-dimensional topology, presents potential as an adsorbent for capturing CO_2_ from moisture-containing gases due to its excellent water, chemical, and thermal stability, attributed to robust metal–ligand bonds, along with its hydrophobic nature that reduces the performance loss due to competitive adsorption of moisture.^15^ Similarly, zirconium(IV)-based UiO-66 MOF is a promising candidate due to its high water stability, structural stability in the presence of strong acids, and ability to withstand high temperatures.^16,17^ These features are mainly attributed to the highly connected network of Zr_6_O4(OH)_4_ clusters with up to 12 terephthalic linkers.^17,18^ The high coordination number of the metal cluster creates steric hindrance, preventing water molecules from clustering around the metal centers, thereby minimizing hydrolysis.^10^ Furthermore, even if some of the metal–ligand bonds break, the framework can withstand more stress before collapsing because there are additional ligands bound to the metal center that provide support. Both ZIF-8 and UiO-66 can be modified to enhance their CO_2_ adsorption capacity and selectivity using different approaches such as hybridization with graphene oxide.^4,19^ Additionally, while the synthesis of most MOFs has been limited to small quantities at the laboratory scale,^20,21^ both ZIF-8 and UiO-66 are among the ones that can be easily produced on a kilogram scale, supporting their industrialization.^22−24^

The utilization of MOFs at large-scale for continuous gas flow is currently primarily hindered by material processing challenges arising from their microcrystalline powder forms, such as dustiness and agglomeration, leading to mass loss, poor mass/heat transfer, and pressure drop within packed beds.^25^ To address these challenges, there is a critical yet challenging need to fabricate millimeter-sized hierarchically porous structured MOFs while preserving or even enhancing their separation performance using a scalable structuring method. Hence, shaping/structuring of MOFs has recently become an intense area of study aimed at developing next-generation adsorption technologies and taking a significant step toward MOF commercialization, facilitating their implementation at the industrial scale, thus unlocking their full potential.^26^

Though classical methods for powder shaping/structuring are applicable to several types of adsorbents and catalysts, their practical applicability to MOFs is often constrained by the mechanical and thermal stability of these materials. Conventional powder shaping techniques, such as pelletization, extrusion, and granulation, often induce irreversible structural changes accompanied by a reduction in the accessible surface area due to the application of high external pressure.^27,28^ Adding binders can lead to performance loss due to pore blocking^22,29^ and/or mass transfer limitations,^30,31^ especially with binders that are nonporous and have poor adsorption properties. Additionally, the high-temperature treatment steps reported in the classical pelletization and extrusion methods^32,33^ and in the emerging 3D-printing methods^34−38^ are unsuitable for MOFs due to the moderate thermal stability of their organic ligands.^39^ Furthermore, some 3D-printing methods require complex preparation processes involving printable inks with different binders or supports at high loadings,^40^ resulting in reduced active MOF loading and direct pore blockage, reducing capture capacity and cyclability performance.

Integrating MOF crystals with porous polymeric matrices under mild preparation conditions represents an alternative strategy to overcome shaping limitations. Combining MOFs with polymers using different methods has been widely studied for the preparation of mixed-matrix membranes (MMMs) for various applications, enabling pre- or postsynthetic modification of MOFs and/or polymers.^41−44^ For instance, the electrospinning method has been employed for the preparation of MOF/polymer composite fiber mats^45,46^ by spinning a MOF/polymer slurry into nanofibers using a high-voltage electric field, however, this method is time-consuming, expensive, and complicated to operate.^47,48^ In contrast, the phase inversion technique often employed is a simple and common approach for preparing stable, flexible, and easily handled MOF/polymer composites by casting suspensions of polymers and preformed MOFs in an aqueous phase or inorganic salt solution.^25^ This versatile and scalable method enables high MOF loading, allows control of MOF loading, and preserves the chemical features and crystalline structure of MOFs,^49,50^ making it an attractive strategy for mass-producing millimeter-sized polymer-based beads with high MOF loading for direct use in fixed-bed columns. In addition to the simple blending of preformed MOFs and polymers, in situ growth of MOFs on prefabricated polymeric beads^51−54^ and layer-by-layer deposition of MOFs^55^ have also been explored, however, these methods often involve a complex and multistep process, apply to a limited subset of MOFs, present difficulty in controlling MOF loading, and typically result in low MOF loading.

Various polymers such as poly(ether sulfone), chitosan, and alginate have been explored as matrices to incorporate different MOFs.^56−59^ Despite enhancing the recyclability, handling, and flexibility of the MOFs, a significant reduction in surface area and porosity (up to 69%),^59^ compared to the parent MOFs, was reported. This reduction highlights the significance of selecting a porous polymer matrix that does not decrease the adsorption of gas molecules through pore blockage or reduction. Instead, it should enhance pore accessibility and preserve the porosity and adsorption properties of the MOF crystals. In this context, macro-porous poly(acrylonitrile) (PAN) polymer matrices,^60,61^ widely used for the preparation of porous membranes, are promising alternatives because they can facilitate gas access pathways to the active sites for molecular uptake and recovery,^50,62−64^ thereby providing the potential to preserve the porosity and gas separation performance of the parent MOFs.

In this work, we present a simple, flexible, and scalable method for structuring moisture-stable and scalable microcrystalline MOFs (UiO-66 and ZIF-8) for direct industrial carbon capture application. Readily processable, millimeter-sized hierarchically porous 3D structured composite beads with ultrahigh MOF loading (∼90 wt %) were prepared by integrating the MOFs with a PAN matrix at mild conditions using the phase inversion technique, which can be easily scaled up for mass production. To examine the potential for structuring the adsorbents without loss of performance and intrinsic properties, crucial for industrial scalability, the morphology, structure, and chemical characteristics of the structured composite beads as well as their CO_2_ capture performance, such as CO_2_ adsorption capacity, CO_2_/N_2_ selectivity, and cyclability under static and dynamic conditions, were evaluated. The facile and scalable structuring method demonstrated here can be applied to other MOF platforms, representing significant progress in the large-scale production of structured MOFs for industrial applications.

## 2 Experimental Section

### 2.1 Materials

Terephthalic acid (H_2_BDC, 99%), poly(acrylonitrile) (PAN, average mol. wt. 150,000), 2-methylimidazole (Hmim, 99%), zinc nitrate hexahydrate (Zn(NO_3_)_2_.6H_2_O, 98%), methanol (CH_3_OH, ≥ 99.8%), poly (vinylpyrrolidone) (PVP, average mol. wt. 360,000), and *N, N-*dimethylformamide (DMF, 99.8%) were purchased from Sigma-Aldrich. Zirconium chloride (ZrCl_4_, anhydrous), hydrochloric acid (HCl, 37%), and dimethyl sulfoxide (DMSO, ≥ 99.9%) were purchased from Merck. These were used for the synthesis and structuring of the MOFs. All chemicals and reagents were of analytical grade and were used as received. For the adsorption experiments, CO_2_ (>99.99%), N_2_ (>99.99%), He (99.99%), and CO_2_/N_2_ (15/85, v v^–1^) mixture gases were supplied by Air Products and Chemicals.

### 2.2 Synthesis of UiO-66 and ZIF-8

UiO-66 was synthesized based on a reported procedure with slight modifications.^24,65^ 570.9 mg of zirconium chloride was dissolved in 122 mL of DMF and stirred for 30 min. In a separate beaker, 399.0 mg of terephthalic acid was dissolved in 122 mL of DMF and stirred for 30 min. The two solutions were mixed under stirring, and 13.5 mL HCl was added to the mixture. The mixture was stirred for 2 h and refluxed for 24 h at 120 °C. The resulting white product was centrifuged, washed several times with DMF and methanol, and dried overnight in a vacuum oven at 60 °C. ZIF-8 was synthesized following a reported procedure.^66,67^ 5.94 g zinc nitrate hexahydrate was dissolved in 200 mL methanol and stirred for 15 min. In a separate beaker, 4.92 g 2-methyl-imidazole was dissolved in 200 mL methanol. The two solutions were mixed under vigorous stirring at room temperature, followed by gentle stirring for 15 h. The resulting white product was centrifuged, washed several times with methanol, and dried for 24 h in a vacuum oven at 60 °C.

### 2.3 Structuring the Adsorbents

UiO-66 and ZIF-8 MOF powders were structured into spherical beads using the phase-inversion technique^50,68,69^ and labeled as MOF@PANy, where y represents the weight loading of PAN in the beads. The preparation of MOF@PAN50 is presented as a representative example. Initially, homogeneous solutions of PAN in DMSO were prepared by adding 0.2 g PAN to 3 mL DMSO and stirring the mixture at 50 °C for 30 min. Then, 0.02 g PVP was added, and the mixture was stirred for 30 min. 0.2 g ZIF-8 or UiO-66 MOF powders were then slowly added, followed by stirring for 30 min. MOF@PAN beads were formed by adding the mixture slurry dropwise into a water coagulation bath. The nonsolvent coagulation bath was replaced with a fresh one, and the beads were left for 30 min and washed with DI water to complete the precipitation of the polymer and to remove DMSO and PVP from the polymer matrix. Finally, the resulting structured composite beads were dried in a vacuum oven at 60 °C overnight. MOF@PAN33 and MOF@PAN90 were prepared using a similar procedure, with 0.4 g MOF and 0.03 g PVP for MOF@PAN33, and 1.8 g MOF and 0.1 g PVP for MOF@PAN90. A similar procedure, but without the addition of MOF crystals, was also followed to prepare neat PAN beads as a reference.

### 2.4 Characterization Techniques

The physicochemical characteristics of the adsorbents were examined using scanning electron microscopy (SEM), X-ray powder diffraction (XRD), Fourier-transform infrared spectroscopy (FTIR), thermogravimetric analysis (TGA), and N_2_ adsorption–desorption isotherms at 77 K. SEM images of the MOF powders and MOF@PAN beads were acquired using a Nova NanoSEM 650 at a working distance of 5 mm and 5–10 kV. To obtain cross-sectional SEM images of the structured composite beads, the beads were immersed in liquid nitrogen, broken into two pieces, and gold-coated before SEM analysis. A D2 PHASER desktop diffractometer was employed to perform XRD analysis of the adsorbents in the 2θ range of 5–50° with Cu–Kα radiation (λ = 1.5406 Å) as the source of X-ray and step size of 0.02° s^–1^. FTIR measurements of the adsorbents were performed using a Bruker Vertex 80v FTIR spectrophotometer equipped with ATR accessories in the frequency range of 4000–400 cm^–1^ with 32 scans and a resolution of 4 cm^–1^. A TA Instruments SDT 650 TG analyzer was used to perform TGA of the adsorbents at a heating rate of 10 K min^–1^ under a N_2_ flow rate of 50 mL min^–1^. The mechanical stability of the structured composite beads was investigated by conducting compression testing using a Discovery Hybrid Rheometer (HR 30) in a parallel plate geometry. A single bead was placed between the plates, with the upper plate moving downward at a constant speed of 5 μm s^–1^. The axial force applied was recorded as a function of the gap between the parallel plates until the beads showed breakage. To account for variations in bead size and the section of the bead compressed, which have an impact on the crushing strength measurements, five representative samples of each structured composite beads were tested. The average crushing strength, measured in N, and the standard deviation were reported to reflect the range and extent of variation among individual beads. A Micromeritics 3Flex Adsorption Analyzer was used to evaluate the textural properties of the adsorbents by performing equilibrium N_2_ adsorption–desorption at 77 K. The measurements were acquired after degassing the samples at 150 °C under vacuum for 6 h. Specific surface area and micro and mesopore size distribution analyses were performed using the Brunauer–Emmett–Teller (BET), Horvath–Kawazoe (HK), and Barret-Joyner-Hallenda (BJH) procedures, respectively.

### 2.5 CO_2_ Capture Performance Evaluation

Both static isotherm measurements and dynamic column breakthrough studies were performed to evaluate the CO_2_ capture performance of the structured composite beads. Using a Micromeritics 3Flex adsorption analyzer, equilibrium CO_2_ and N_2_ adsorption isotherms were obtained up to 1 bar under static conditions. The Micromeritics 3Flex adsorption analyzer coupled with a manifold equipped with a vapor source was also utilized to measure the water adsorption isotherm of the adsorbents at a relative pressure range of 0 to 0.4. The results for the structured composite beads are reported per total mass of the MOF@PAN beads (mmol g^–1^). Before analysis, all the samples were treated at 150 °C under vacuum for 6 h. The cyclability of the adsorbents was investigated by performing 10 continuous cycles of vacuum swing adsorption (VSA) at 25 °C, involving CO_2_ adsorption at 1 bar followed by adsorbent regeneration via pressure reduction without external heating. The Clausius–Clapeyron (eq 1)^5,70^ was used to calculate the isosteric heat of CO_2_ adsorption (Δ*H*_ads_) at constant loading using CO_2_ adsorption isotherms collected at 25, 35, and 45 °C.
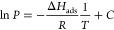
1where *R* is the ideal gas constant, *T* is the temperature, and *C* is an integration constant. The CO_2_/N_2_ selectivity of the adsorbents for CO_2_/N_2_ gas mixtures was estimated using eq 2.^5^
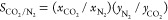
2where *y* represents the mole fraction of the gas species in the bulk gas phase, while *x* denotes the corresponding mole fraction in the adsorbed phase.

The dynamic CO_2_ adsorption performance of the structured composite beads was evaluated using dynamic breakthrough experiments with a mixSorb S from 3P Instruments. These experiments are designed to assess the CO_2_ capture performance of adsorbents under continuous flow conditions, which more closely mimic practical application scenarios. In these studies, the adsorbent is subjected to a continuous flow of CO_2_-containing gas mixture, and the breakthrough curve is recorded by monitoring the concentration of gases in the effluent to determine when the adsorbent becomes saturated and how effectively it captures CO_2_ over time. In our study, the experiments were conducted using a feed gas with a CO_2_/N_2_ volume composition of 15:85, with helium as carrier gas, at 25 °C, a flow rate of 30 mL min^–1^, and a total pressure of 1.25 bar. The gases leaving the column were analyzed using a mass spectrometer (CIRRUS.3 from MKS). Before the analysis, the structured composite beads were activated by He purging at 150 °C for 6 h. To account for the dead volume and response time of the spectrometer, blank breakthrough experiments using glass beads were performed and the CO_2_ and N_2_ adsorption from these experiments was then subtracted from the measurements of the structured composite beads. The dynamic recyclability and stability of the structured composite beads were assessed by performing 10 continuous breakthrough cycles at 25 °C. Following each breakthrough adsorption step, CO_2_ desorption was achieved by purging with helium at a flow rate of 100 mL min^–1^ for 2 h, without external heating or pressure reduction. The amount of gas adsorbed (*q*_*ads*_) during the breakthrough experiments up to any time instant t was calculated from mass balance using the Software for mixSorb S using eq 3.^38^
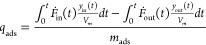
3where and  represent the total volumetric flow rate of the gas mixture at the inlet and outlet any instant *t*, respectively. *m*_ads_ is the dry mass of the adsorbent, *V*_*m*_ is gas molar volume, and *y*_in_(*t*) and *y*_out_(*t*) denote the gas mole fraction at the inlet and outlet at any instant time, respectively.

## 3 Results and Discussion

### 3.1 Fabrication and Characterization of the Structured Adsorbents

**Figure 1:**
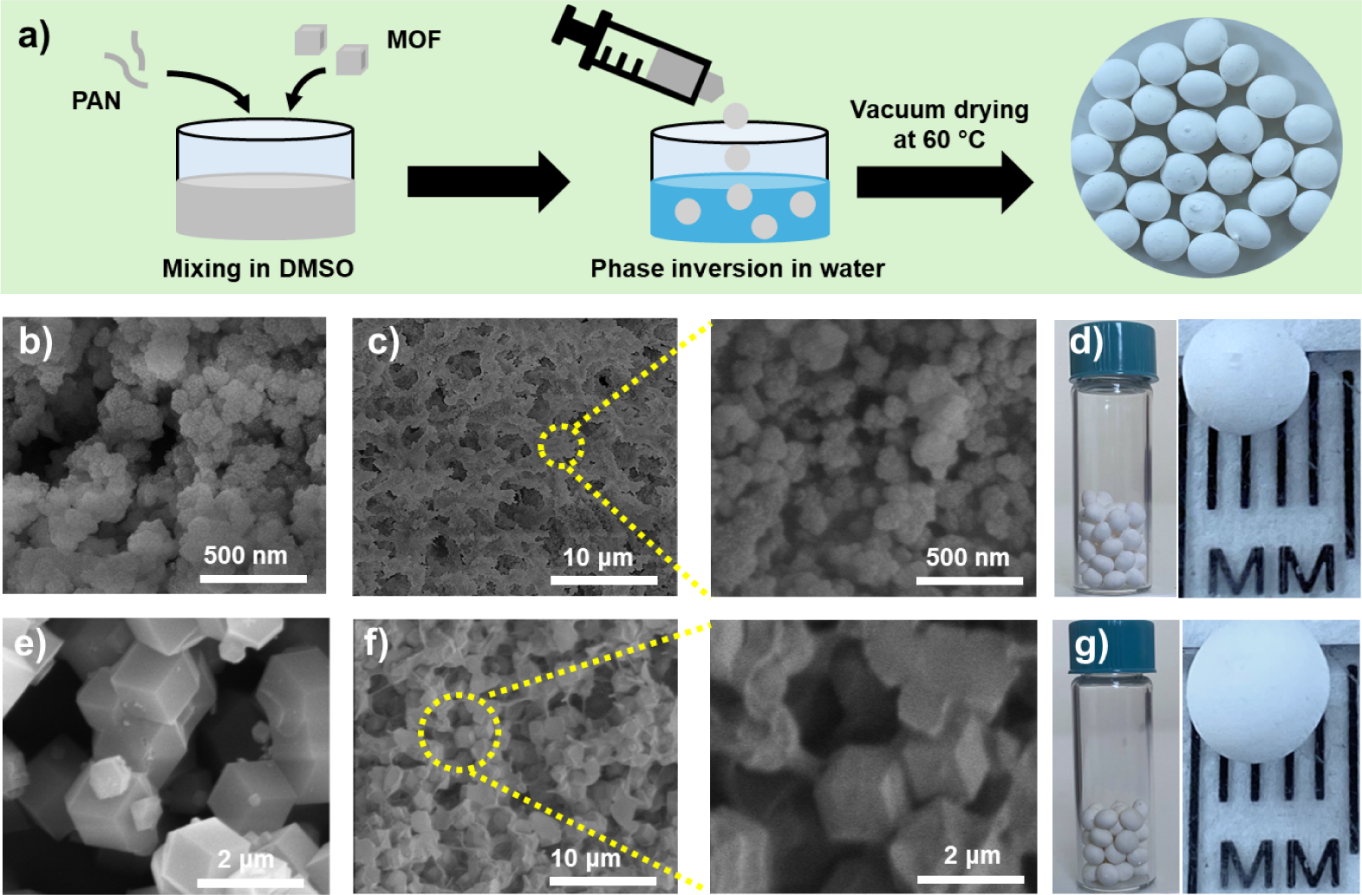
(a) Schematic illustration of the structuring process of MOFs using the phase inversion method. SEM images of (b) UiO-66 powder and (c) inner cross-section of UiO-66@PAN10 bead. (d) Photograph of the UiO-66@PAN10 bead. SEM images of (e) ZIF-8 powder and (f) inner cross-section of ZIF-8@PAN10 bead. (g) Photograph of the ZIF-8@PAN10 bead.

The phase inversion method employed to structure the adsorbents, as described in detail in the experimental section, is illustrated in Figure 1a. UiO-66 or ZIF-8 MOF crystal powders were synthesized and mixed with PAN in DMSO, followed by phase inversion taking place in water as the nonsolvent, leading to the solidification of the droplets into MOF@PAN beads. As a higher weight loading of PAN would dilute the MOF powders, we aimed to maximize the loading of the MOF powders, achieving a maximum of 90 wt % MOF loading, beyond which stable MOF beads could not be formed by the phase inversion method. Structured adsorbents containing 50 and 67 wt % MOF loadings were also prepared for comparison. The respective structured adsorbents are denoted as MOF@PANy, where y represents the weight loading of the PAN polymer matrix in the beads.

The structured UiO-66 and ZIF-8 adsorbents have spherical shape with diameters ranging from 3 to 4 mm, as shown in Figure 1d,g, making them suitable for direct loading into packed columns for practical CO_2_ capture applications. The morphology and internal structure of the beads were examined by SEM. The SEM images presented in Figure 1 show that the morphology of the UiO-66 and ZIF-8 crystals in the corresponding MOF@PAN beads are similar to those of the parent UiO-66 and ZIF-8 crystals, indicating that the structuring process has a negligible impact on the morphology of the individual UiO-66 and ZIF-8 crystals. Furthermore, the cross-sectional SEM images of the UiO-66@PAN10 (Figures 1c and S1) and ZIF-8@PAN10 beads (Figures 1f and S2) reveal a complex, three-dimensional porous network structure exhibiting long, finger-like pores of 10–20 μm in width that extend from just beneath the surface of the bead toward its inner section, similar to structures observed in mixed matrix membranes (MMMs) prepared using the phase inversion method.^71−73^ Additionally, the inner section displays a well-interconnected network of micron-sized voids and contains individual MOF crystal particles dispersed within the composite PAN polymer matrix. This hierarchical porosity is expected to ensure that the MOF crystal particles remain accessible and to enhance the molecular transport of CO_2_ within the porous section toward the embedded MOF crystals and vice versa, thereby facilitating both adsorption and desorption. However, the presence of a rough outer surface layer with smaller pores may provide additional mass transfer resistance for gas molecules. Nonetheless, kinetic data (Figure 5a,b) reveal that the overall kinetics of sorption for the structured composite beads are comparable to those of the respective MOF powders, indicating negligible mass transfer resistance of the outer surface layer.

**Figure 2:**
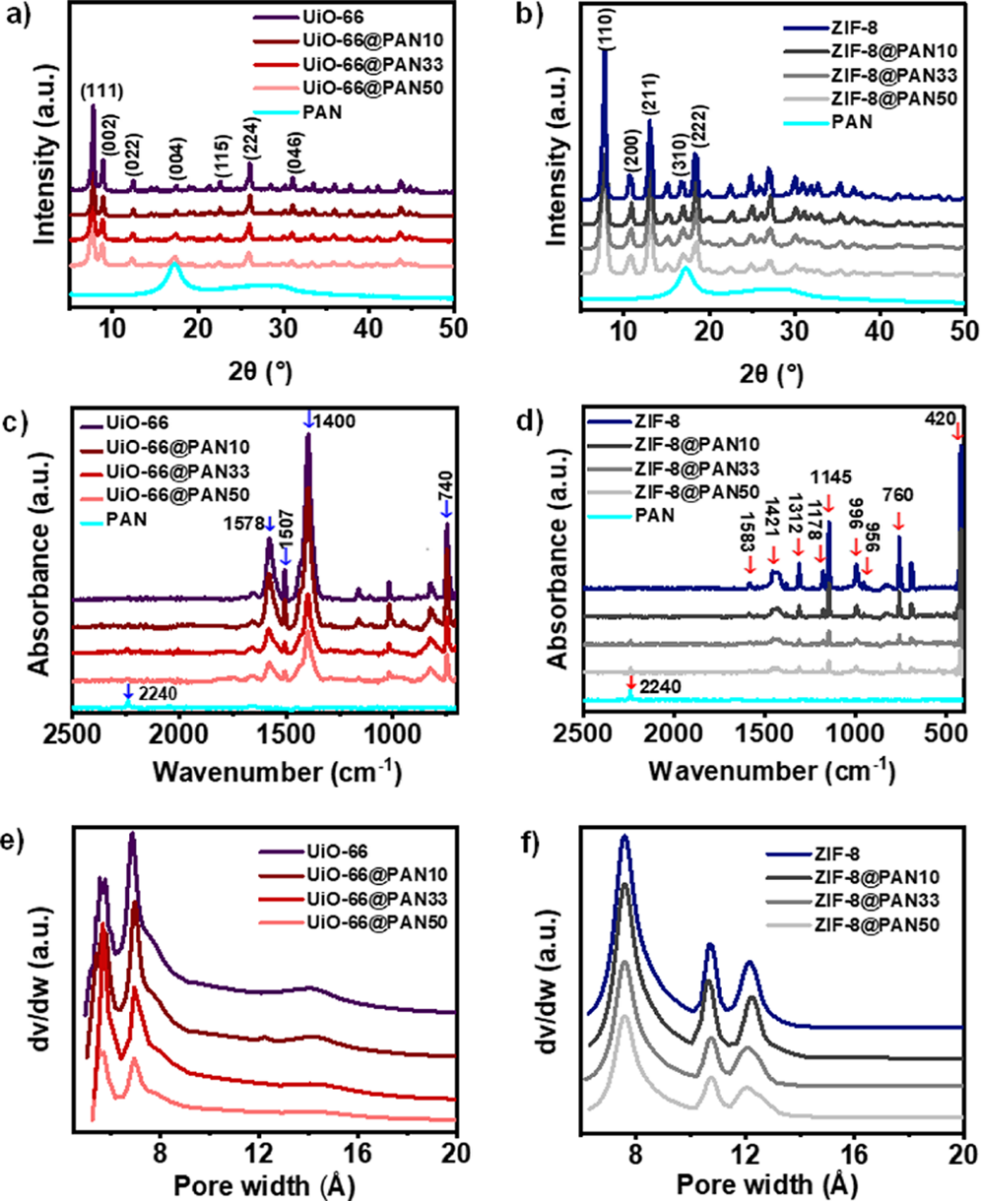
XRD patterns, FTIR spectra, and micropore size distributions of (a,c,e) UiO-66@PAN and (b,d,f) ZIF-8@PAN beads, respectively, in comparison to the MOF powders.

The effect of the structuring process on the crystalline nature of the UiO-66 and ZIF-8 particles was investigated by XRD analysis of the adsorbents before and after structuring, as shown in Figure 2a,b. The peaks of UiO-66@PAN and ZIF-8@PAN beads are in agreement to those of the UiO-66 and ZIF-8 powders, respectively, confirming the preservation of the MOFs’ crystalline structure after integration into the PAN matrix. Thermal analysis demonstrates the robust thermal stability of the PAN matrix, with decomposition temperatures exceeding 300 °C, as evidenced by the TGA curves presented in Figure S3. Furthermore, the decomposition onset temperature in MOF@PAN beads is shifted to higher temperatures compared to that of MOF powders, indicating enhanced thermal stability. Additionally, the nonhydrophilic nature of PAN is indicated by the absence of weight loss associated with physically adsorbed water molecules, which typically occurs up to 150 °C. This property makes PAN a suitable matrix for developing structured adsorbents to capture CO_2_ from moisture-containing mixtures. The hydrophobic nature of ZIF-8 is also evident from the TGA curves, while the hydrophilicity of UiO-66@PAN beads is suppressed compared to that of the parent powder due to the incorporation of the PAN matrix.

FTIR spectra of UiO-66 and ZIF-8 before and after structuring were collected (Figure 2c,d) to evaluate the impact of structuring on the chemical characteristics of the adsorbents. The FTIR spectra of UiO-66@PAN and ZIF-8@PAN beads are analogous to those of UiO-66 and ZIF-8 powders, respectively, and the intensity of the bands increases with increasing loading of the MOF powders, indicating that the chemical characteristics of the individual UiO-66 and ZIF-8 particles are preserved in the MOF@PAN beads. The structured composite beads exhibit a band at 2240 cm^–1^, corresponding to the -C ≡ N stretching of PAN,^74^ indicating the presence of PAN in the MOF@PAN beads. Notably, no new bands appeared in the FTIR spectra of the MOF@PAN beads, suggesting that physical interactions between the MOFs and PAN matrix play a key role in the formation of the structured adsorbents.

In practical applications, structured adsorbents are often packed into large columns where they experience pressure from adsorbents placed on top and from the gas flowing through the column, which can lead to breakage and compaction, leading to reduced performance, mass transfer limitations, and pressure drops. Therefore, understanding the mechanical properties of the structured composite beads is crucial for their deployment in practical applications. The mechanical stability of the structured composite beads was evaluated and compared with neat PAN beads through compression testing (Figure S4 c–f). Pure PAN beads, i.e., without the MOF phase, exhibited high crushing strength of 49.9 ± 0.02 N. ZIF-8@PAN10 and ZIF-8@PAN33, comprising 10 and 33 wt % PAN, respectively, showed crushing strengths of 7.2 ± 0.12 N and 11.4 ± 0.85 N, while UiO-66@PAN10 and UiO-66@PAN33 exhibited strengths of 3.5 ± 0.56 N and 8.7 ± 0.21 N, respectively. These results indicate that a higher polymer loading enhances the crushing strength of the structured composite beads, yet, the obtained crushing strengths of the structured adsorbents in this study are comparable to or higher than those reported for other structured MOF composites beads, such as ZIF-67@chitosan beads with 33.3 wt % polymer loading (1.57 ± 0.06 N),^75^ ZIF-8@PVFM beads with 15 wt % polymer loading (3.09 ± 0.97 N),^76^ and commercial adsorbents beads widely used for fixed-bed applications like zeolite 3A beads (8.03 ± 2.67 N).^76^ The structural integrity of the UiO-66@PAN10 and ZIF-8@PAN10 beads under gas pressure was also studied by exposing the samples to 10 bar of N_2_ for 16 h using Hiden Isochema Intelligent Gravimetric Analyzer (IGA). The pictures of the structured composite beads after gas exposure, depicted in Figure S4a,b, show no visual fractures in the beads, indicating their ability to maintain their structure under high-pressure conditions. These findings indicate that the resulting structured composite beads are mechanically robust for practical CO_2_ capture applications.

N_2_ adsorption–desorption measurements were conducted at 77 K to evaluate the impact of structuring on the porosity characteristics of UiO-66 and ZIF-8 particles. The N_2_ adsorption–desorption results and the associated textural properties for the structured composite beads are reported per unit total mass of the MOF@PAN beads. The N_2_ adsorption–desorption isotherms of the structured composite beads (Figure S5) show that UiO-66@PAN and ZIF-8@PAN beads, similar to their respective MOF powders, are predominantly microporous, as evidenced by the steep rise in N_2_ adsorption capacity at very low relative pressures. Table S1 presents the porosity parameters of the adsorbents, including the BET specific surface area, total pore volume, micropore volume, and average micropore diameter, as calculated from the isotherms. A comparison of the surface area and pore volume of ZIF-8 and UiO-66 powders with those of similar MOFs reported in the literature is also included in the Supporting Information. The micropore size distributions, estimated using the HK method, are shown in Figures 2e,f and S5c,d. The results show that the micropore size distributions of the structured adsorbents are similar to those of their corresponding MOF powders, with UiO-66@PAN beads displaying peaks centered at 6 and 7 Å, similar to the UiO-66 powder, and ZIF-8@PAN beads showing peaks centered at 7.5 Å, 10.7 Å, and 12.1 Å, similar to the ZIF-8 powder. The amount of N_2_ adsorbed and BET surface areas of the MOF@PAN beads increase with increasing MOF loading (Figures S5 and S6). These observations indicate the contribution of the MOF particles to the porosities of the structured composite beads and provide evidence that the porosity characteristics of the UiO-66 and ZIF-8 crystals were retained after the structuring process. Meanwhile, the individual MOF crystals are dispersed within the PAN matrix, which facilitates the access and exposure of their active sites to CO_2_ molecules. Furthermore, the pore size distributions in the mesopore range (20–500 Å), derived using the BJH method (Figure S7a), reveal the presence of mesopores in the UiO-66@PAN beads, which could be attributed to interstitial voids between nanosized UiO-66 particles, as indicated by the high N_2_ adsorption and hysteresis loop at high p/p_0_ (Figure S5a) and reported elsewhere.^77,78^ In contrast, the mesopore size distributions of the ZIF-8@PAN beads (Figure S7b), prepared with ZIF-8 particles of an average size of 1 μm, show minimal mesoporosity contribution, as also reflected by a small difference between the total pore volume and micropore volume. However, it has been demonstrated that both structured ZIF-8 and UiO-66 adsorbents possess a macroporous network with interconnected micron-sized voids, indicating the presence of hierarchical porosity.

According to the data in Table S1, pure PAN beads, without the MOF phase, exhibit a negligible surface area and pore volume, measuring 4.6 m^2^ g^–1^ and 0.08 cm^3^ g^–1^, respectively. On the other hand, UiO-66@PAN10, the bead with 90 wt % UiO-66 loading, exhibits the highest specific surface area and pore volume among the structured UiO-66 adsorbents, measuring 1130 m^2^ g^–1^ and 0.69 cm^3^ g^–1^, respectively. Similarly, among the structured ZIF-8 adsorbents, ZIF-8@PAN10 exhibits the highest specific surface area and pore volume, measuring 1431 m^2^ g^–1^ and 0.62 cm^3^ g^–1^, respectively. The maintenance of high specific surface area and pore volume of the structured adsorbents can be attributed to several factors, including the shaping process and the choice of material used. First, the shaping process was conducted under mild conditions, with temperatures below 50 °C and without applying high pressure to the MOF crystals. These conditions help preserve the chemical stability and crystallinity of the MOFs, whereas extreme conditions, such as high pressure, often lead to a loss of crystallinity and porosity.^79^ Additionally, the choice of the composting polymer is crucial because polymers can potentially block or penetrate MOF pores, which would reduce surface area and porosity.^80^ In the present work, a high molecular weight, macroporous PAN matrix^60,61^ was used, which limits polymer penetration and prevents pore blockage, thereby helping to maintain the porosity of the MOFs. Furthermore, the phase inversion process using a high molecular weight PVP as a pore-forming agent creates a macroporous network with interconnected macrovoids in which the individual MOF particles are dispersed throughout the polymer matrix, ensuring that the MOF pores remain accessible. Overall, the combination of using a high molecular weight, macroporous PAN matrix, and applying mild shaping conditions contributes to preserving the MOF’s structural integrity and porosity. This approach is highly attractive for CO_2_ capture applications when compared to traditional methods, such as pelletization and extrusion, as well as other structured MOF-polymer composites.

For instance, ZIF-8 pellets prepared using mechanical compression at 0.9 and 1.2 GPa exhibited irreversible textural and structural changes, including amorphization and surface area reduction (50.1% and 57.3%, respectively).^79^ Similar issues were observed in other structured MOFs subjected to high pressure.^28,81−84^ UiO-66 pellets prepared with 10 wt % sucrose as a binder exhibited a BET specific surface area of 674 m^2^ g^–1^, representing a 50% reduction compared to the expected surface area based on UiO-66 loading.^29^ Shaping UiO-66_COOH with 5.5 wt % of silicon resin through extrusion resulted in a 40% loss of specific surface area,^85^ both cases attributed to pore blockage. Similarly, MOF-177-TEPA-20% pellets prepared with 4% poly (vinyl butyral) (PVB) binder and 3.7 kN m^–2^ pressure showed reduced adsorption capacity due to pore blockage by the binder, reduced crystallinity due to the applied pressure and dense pellet formation.^86^ Shaping MIL-53 using 15 wt % poly(vinyl alcohol) (PVA) binder through mixing, heating at 190 °C, and crushing resulted in a 32% reduction in surface area, which can be attributed to pore blockage by the polymer and the dense structure obtained.^87^ Other composting polymers, without the application of external high pressure, have also been investigated for shaping MOFs. For instance, UiO-66@chitosan composites containing MOF loadings of 66.7 wt %^56^ and 50 wt %^57^ were prepared through freeze-drying a mixture of chitosan and UiO-66 in an acetic acid aqueous solution. These structured composites exhibited BET specific surface areas of 338 and 122 m^2^ g^–1^, respectively, resulting in an approximately 50% reduction compared to the expected surface area based on the UiO-66 loading. The decrease in surface area was attributed to the breakdown of the UiO-66 crystal structure by the NaOH solution used to remove residual acetic acid,^56^ as well as pore blockage by chitosan and pore penetration and/or pore blockage by glutaraldehyde molecules added as cross-linking agent.^57^ Similarly, HKUST-1@ poly(ether sulfone) composite beads, with 72% MOF loading showed a BET specific surface area of 237 m^2^ g^–1^, indicating a 69% reduction relative to the surface area expected based on HKUST-1 loading.^59^ ZIF-8@aliginate and HKUST-1@alginate composites exhibited surface areas of 563 and 19 m^2^ g^–1^, respectively,^58^ which are very low compared to the reported surface areas of pristine ZIF-8 (1340 m^2^ g^–1^)^88^ and pristine HKUST-1 (1379.87 m^2^ g^–1^).^5^

In conclusion, the use of a mechanically robust, chemically stable, and macroporous PAN matrix to structure MOFs through the simple and scalable method under mild conditions demonstrated here is effective in preserving the porosity characteristics and crystallinity of the individual MOF crystals compared to conventional methods such as pelletization and extrusion. It is also more effective than alternative composting polymers such as sucrose, chitosan, and alginate. Furthermore, the method has considerable potential for the large-scale manufacturing of structured adsorbents due to its simplicity, speed, and potential for automation. For instance, an automated peristaltic pump with an attached needle can be used to continuously add the MOF/polymer slurry dropwise into a coagulation bath, enabling the continuous production of structured adsorbents. Assuming a constant count rate of 120 slurry drops per min, approximately 7200 beads can be produced in 1 h using a single dropping point, which corresponds to around 1.7 kg per day. Both the mass and production rates of the structured adsorbents can be increased by employing several dropping points, indicating that the method is easily scalable and suitable for the large-scale production of structured adsorbents.

### 3.2 Gas Adsorption Evaluation of the Structured Beads

**Figure 3:**
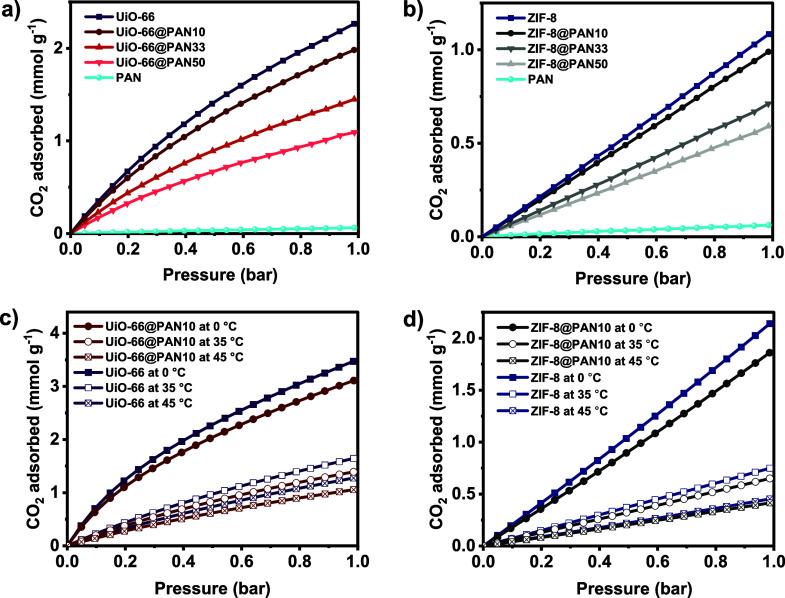
CO_2_ adsorption isotherms at 25 °C for (a) UiO-66@PAN and (b) ZIF-8@PAN beads, compared to pure PAN beads and the corresponding pure MOF powders. CO_2_ adsorption isotherms at 0 °C, 35 °C, and 45 °C for (c) UiO-66@PAN10 and (d) ZIF-8@PAN10 and their corresponding MOF powders. Data presented per unit of total mass of the MOF@PAN beads.

The gas adsorption properties of the structured composite beads were evaluated and compared with those of the parent MOF powders and neat PAN beads by collecting the CO_2_ and N_2_ adsorption isotherms in the pressure range of 0 to 1 bar, first under static conditions. The gas adsorption results for the structured adsorbents are reported in mmol per total mass of the beads (mmol g^–1^). Figure 3a,b present the equilibrium adsorption isotherms of CO_2_ at 25 °C. The results demonstrate that the CO_2_ uptake of the structured composite beads increases with pressure, similar to the behavior observed in the respective MOF powders, with no saturation observed within the investigated pressure range. This indicates that the adsorbents can adsorb more CO_2_ as pressure increases. At 25 °C and 1 bar, the CO_2_ adsorption capacity of UiO-66 and ZIF-8 in powder form are 2.2 and 1.1 mmol g^–1^, respectively, while pure PAN beads (without MOF) exhibit a negligible CO_2_ capacity of 0.06 mmol g^–1^. The higher CO_2_ adsorption capacity of UiO-66 compared to ZIF-8 is attributed to the greater affinity of UiO-66 for CO_2_, evidenced by its higher heat CO_2_ of adsorption discussed below. As shown in Figure S8b–e, in UiO-66, CO_2_ binds through hydrogen bonding with μ3-OH groups located within tetrahedral pores, through dispersion forces within its confined pore environment, and by binding to open zirconium sites formed due to defects or missing linkers.^89,90^ In contrast, CO_2_ in ZIF-8 primarily binds via dispersion forces within the small pores formed by the ligands^91,92^ (see Figure S8a).

The CO_2_ adsorption capacity of the MOF@PAN beads increases with the weight loading of the MOFs. For example, upon structuring with 90 wt % MOF loading, UiO-66@PAN10 and ZIF-8@PAN10 exhibit CO_2_ uptakes of 2.0 and 1.0 mmol g^–1^, respectively. To ensure a fair comparison between the CO_2_ capacity of the MOFs integrated into the PAN matrix and the pristine MOF powders, the capacities of the MOF@PAN beads were normalized relative to the amount of the MOF phase. In this case, UiO-66@PAN10 and ZIF-8@PAN10 exhibit CO_2_ adsorption capacities of 2.2 mmol g^–1^ and 1.1 mmol g^–1^, respectively, at 25 °C and 1 bar, i.e., per g of MOF only. These values are consistent with the CO_2_ capacities of the pure MOF powders, indicating that PAN has a negligible influence on the CO_2_ capacity and primarily serves as a support and dispersion medium for the MOF crystals. Furthermore, this indicates that the structuring process not only maintained the crystal structure, chemical properties, and porosity characteristics but also retained the CO_2_ adsorption capacity of the individual UiO-66 and ZIF-8 particles.

In contrast, conventional powder shaping techniques, such as pelletization and extrusion, often result in both reduced surface area and CO_2_ adsorption performance. For instance, MOF-177 pellets prepared with 4 wt % poly (vinyl butyral) binder under a compression pressure of 3.7 kN m^–2^ exhibited a 30% decrease in CO_2_ adsorption capacity (from 0.8 to 0.55 mmol g^–1^ at 25 °C and 1 bar) compared to the corresponding MOF-177 powder.^86^ This reduction was attributed to pore blockage by the binder and reduced crystallinity due to the applied pressure. Similarly, the CO_2_ adsorption capacity of MIL-101 decreased by 35% after pelletization due to a decreased surface area,^93^ while CuBTC showed a 42% decrease in CO_2_ uptake capacity and reduced crystallinity after pelletization conducted at 3.7 kN m^–2^.^94^ Notably, the equilibrium CO_2_ adsorption capacity of the structured composite beads with 90 wt % MOF loading prepared in the present work are superior or comparable to many reported structured adsorbents, as shown in the comparison Table S2. For instance, the CO_2_ adsorption capacity of ZIF-8@PAN10 at 1 bar and 298 K (1.0 mmol g^–1^) surpasses that of reported ZIF-8-based structured composites such PI/ZIF composite aerogels (0.4 mmol g^–1^),^95^ chitosan/ZIF-8 composites beads (0.56 mmol g^–1^),^96^ nanocellulose/ZIF-based foams (0.62 mmol g^–1^)^97^ and ZIF-67/CS2:1 cryogels (0.76 mmol g^–1^).^75^ Similarly, UiO-66@PAN10, with a CO_2_ uptake capacity of 2.0 mmol g^–1^ at 298 K and 1 bar, outperforms several structured composites, including MOF-177/PVB pellets (0.55 mmol g^–1^),^86^ UTSA-16(Co)-cordierite monolith (1.1 mmol g^–1^),^98^ HKUST-1@Torlon monolith (1.2 mmol g^–1^),^99^ and MOF-74(Ni)-cordierite monolith (1.7 mmol g^–1^),^98^ in addition to the above-mentioned ZIF-8-based structured composites. Furthermore, as presented in Table S2, the equilibrium CO_2_ adsorption capacity of UiO-66@PAN10 at 0.15 bar and 298 K is comparable to or superior to that of several structured composites, except for the SIFSIX-3-Cu and MOF-74 based structured adsorbents, which exhibit higher CO_2_ adsorption capacities at lower pressures. However, the exceptional moisture stability of the ZIF-8 and UiO-66 used in the present work provides a practical advantage over several moisture-sensitive MOFs, such as SIFSIX-3-Cu, MOF-74, and HKUST-1, which lose their structure and performance when exposed to moisture.^10−12,14,100^

UiO-66@PAN10 and ZIF-8@PAN10, the beads with the highest MOF loading and CO_2_ adsorption capacity, were selected for further investigation. The CO_2_ adsorption isotherms of the structured composite beads were also evaluated at 0 °C, 35 °C, and 45 °C to investigate the effect of temperature on CO_2_ uptake (Figure 3c,d). The results show that the CO_2_ uptake increases with decreasing temperature, suggesting that physisorption is the primary mechanism of CO_2_ capture of the structured beads. Initially, CO_2_ is transported from the bulk gas phase to the surface of the structured composite beads through the PAN matrix pores, and then it diffuses into the MOF micropores where it is adsorbed onto the active sites. In this case, the MOF phase primarily contributes to the CO_2_ uptake, whereas PAN contributes minimally due to its low affinity for CO_2_. However, the interconnected macropores of the PAN matrix in the structured composite beads act as fast transport channels, allowing CO_2_ molecules to easily reach the MOF active sites. Furthermore, the results confirm that UiO-66@PAN10 and ZIF-8@PAN10 beads maintain the CO_2_ adsorption capacities of the corresponding MOF powders under all investigated operating conditions. For instance, UiO-66@PAN10 and ZIF-8@PAN10 beads exhibit CO_2_ adsorption capacities of 3.2 mmol g^–1^ and 2.2 mmol g^–1^, respectively, at 0 °C and 1 bar, which are 90% of the CO_2_ uptake capacity of the corresponding parent MOFs, in agreement with the weight percent loading of the corresponding MOF phases in the beads.

**Figure 4:**
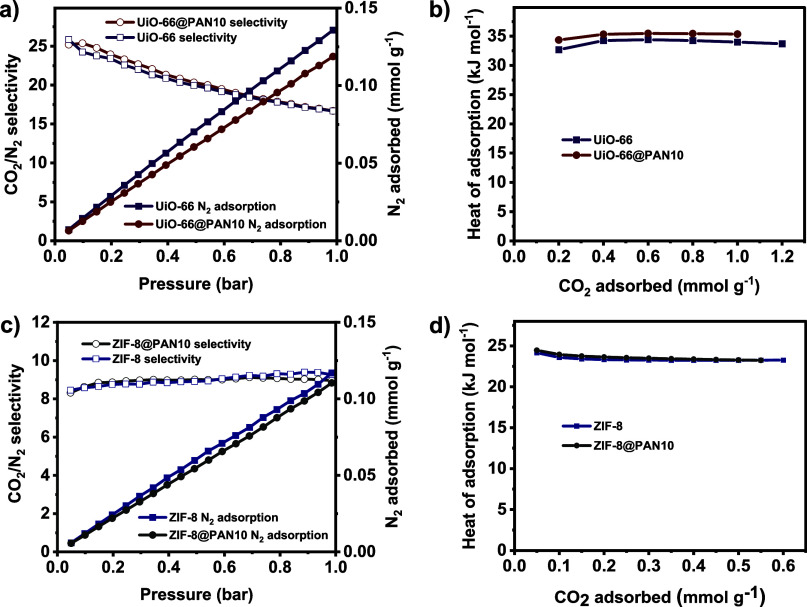
N_2_ adsorption isotherms and ideal CO_2_/N_2_ selectivity at 25 °C, and heat of CO_2_ adsorption for (a,b) UiO-66@PAN10 and (c,d) ZIF-8@PAN10, compared to the corresponding MOF powders. Data presented per unit of total mass of the MOF@PAN beads.

The CO_2_/N_2_ separation performance of the structured adsorbents was assessed by estimating their CO_2_/N_2_ selectivity based on the experimental CO_2_ and N_2_ adsorption isotherms measured at 25 °C and pressures of up to 1 bar. Figure 4a,c illustrate that the UiO-66@PAN10 and ZIF-8@PAN10 have low N_2_ uptake capacities of 0.12 and 0.11 mmol g^–1^, respectively, at 1 bar and 25 °C, i.e., considerably lower than the corresponding CO_2_ adsorption capacities. As a result, at 25 °C and pressures of 0.15 and 1 bar, the UiO-66@PAN10 exhibits ideal CO_2_/N_2_ selectivity of 24.8 and 16.7, respectively, and the ZIF-8@PAN10 displays ideal CO_2_/N_2_ selectivity values of 8.9 and 9.0, respectively. The ideal CO_2_/N_2_ selectivity values of the structured composite beads are analogous to those of the corresponding MOF powders (Figure 4), indicating negligible influence of the PAN polymer matrix on the CO_2_/N_2_ separation performance of the MOF particles. The structured composite beads, in addition to their suitable size and shape, which enable their direct loading into packed beds for practical CO_2_ capture applications, also hold potential for pre- or postsynthesis modification of MOFs, such as hybridization with various materials such as graphene oxide that can also be incorporated into the polymer matrix to increase the CO_2_ adsorption capacity and selectivity of the MOFs.^19,101^ These characteristics suggest that these structured composite beads hold significant promise for industrial applications compared to their corresponding MOF powders.

The isosteric heats of CO_2_ adsorption for the structured composite beads were calculated, utilizing the CO_2_ adsorption isotherms collected at different temperatures (Figure 3), to assess the CO_2_ adsorption mechanism and the strength of the interactions between the structured composite beads and CO_2_. Figure 4b,d show that the heat of adsorption of the structured composite beads remains almost constant with increasing surface coverage, suggesting homogeneous surface adsorption sites for CO_2_ molecules. Both UiO-66@PAN10 and ZIF-8@PAN10 beads exhibit isosteric heats of CO_2_ adsorption comparable to those of the respective MOF powders, indicating that the interaction energy between the individual MOF particles and CO_2_ molecules in the MOF@PAN beads was not distracted by the presence of PAN. The heat of adsorption of UiO-66@PAN10 (34.3–35.3 kJ mol^–1^) and ZIF-8@PAN10 beads (22.9–24.4 kJ mol^–1^) are within the physisorption range (25–50 kJ mol^–1^)^3,102^ indicating physical interactions between the structured composite beads and CO_2_, which facilitates regeneration of the structured composite beads. The heat of CO_2_ adsorption values of the structured composite beads are comparable to those of other reported analogous physical adsorbents, such as MOF-74 (Ni)-cordierite monolith (31–35 kJ mol^–1^),^98^ UTSA-16(Co)-cordierite monolith (29–33 kJ mol^–1^),^98^ MOF-74(Mg)@SBA-15 (29.9–30.3 kJ mol^–1^),^103^ HKUST-1 @GO (26 kJ mol^–1^),^104^ and HKUST1 @aminated GO (22 kJ mol^–1^).^105^

**Figure 5:**
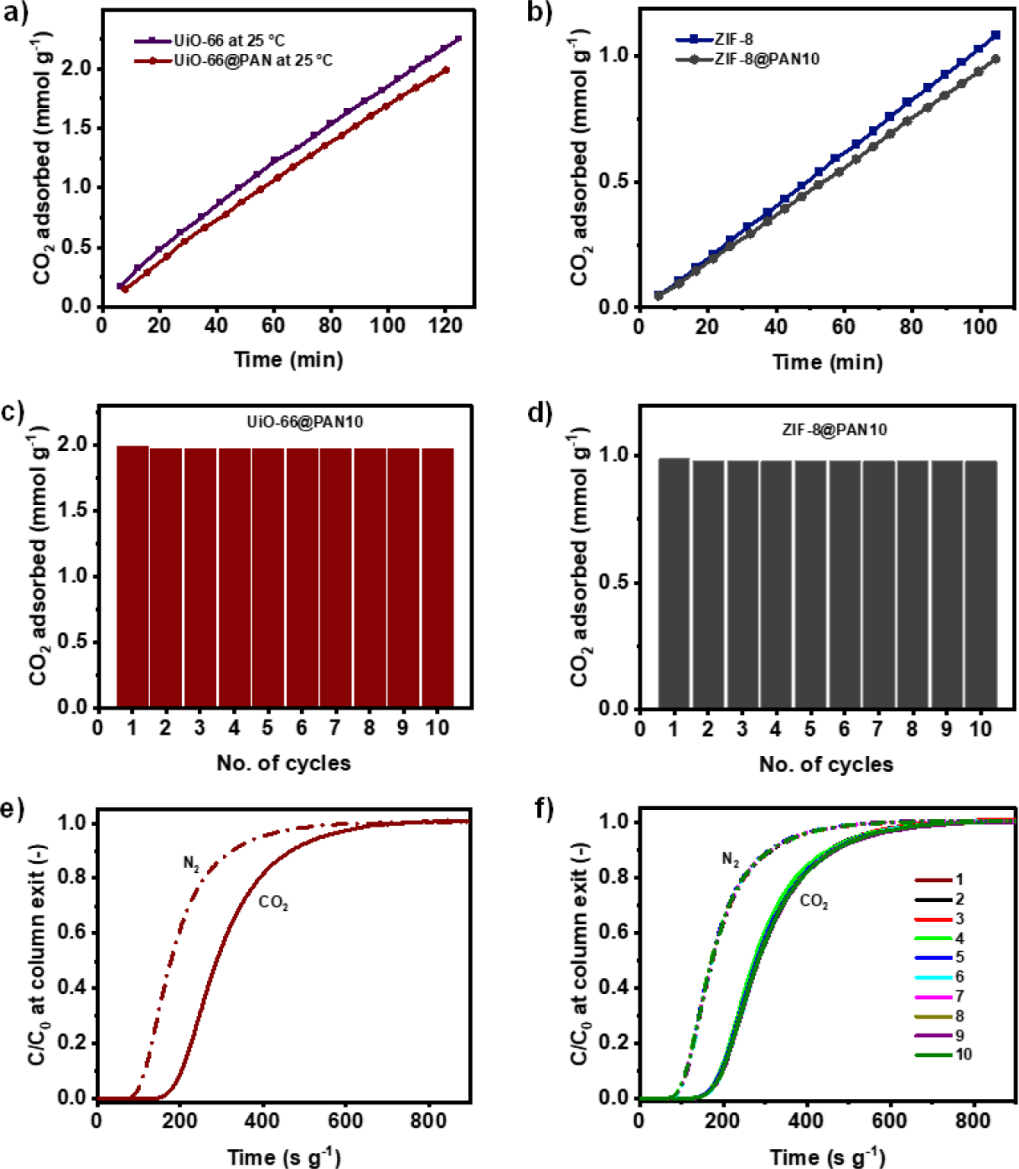
CO_2_ adsorption kinetics for (a) UiO-66@PAN10 and (b) ZIF-8@PAN10 beads at 25 °C and 1 bar, compared to the respective MOF powders. VSA-based cyclic performance at 1 bar and 25 °C for (c) UiO-66@PAN10 and (d) ZIF-8@PAN10. (e) Experimental CO_2_ and N_2_ breakthrough curves and (f) dynamic cyclic performance for 10 breakthrough cycles of UiO-66@PAN10 using a gas mixture with a CO_2_/N_2_ volume ratio of 15/85 at 25 °C, a flow rate of 30 mL min^–1^, and a total pressure of 1.25 bar with helium as the carrier gas. Helium purging was used without external heating for the regeneration of the adsorbent bed after each cycle. Data presented per unit of total mass of the MOF@PAN beads.

The CO_2_ adsorption and desorption kinetics of the structured adsorbents, which are crucial parameters that determine the cycle time, number and size of an adsorber column, and amount of adsorbent for a cyclic adsorption process,^3,106^ were investigated at 25 °C and pressures of up to 1 bar. Figure 5a,b show the adsorption kinetics of the structured composite beads and their respective MOF powders, while Figure S9a,c show the desorption kinetics. The average slopes of the equilibrium kinetic curves were used to estimate the average adsorption and desorption rates. Accordingly, the CO_2_ adsorption rates of UiO-66@PAN10 and ZIF-8@PAN10 beads are 0.0163 and 0.0096 mmol g^–1^ min^–1^, respectively. For a direct comparison of the CO_2_ adsorption kinetics between the MOFs integrated into the PAN matrix and the pristine MOF powders, the CO_2_ adsorption kinetics of the MOF@PAN beads were normalized relative to the amount of the MOF phase. In this case, the estimated CO_2_ adsorption rates, when reported per g of MOF only, for UiO-66@PAN10 (0.0181 mmol g^–1^ min^–1^) and ZIF-8@PAN10 (0.0106 mmol g^–1^ min^–1^) are greater than the corresponding rates observed for the pristine UiO-66 (0.0171 mmol g^–1^ min^–1^) and ZIF-8 powders (0.0104 mmol g^–1^ min^–1^). Regarding the desorption kinetics, a significant parameter for the design and scheduling of cyclic CO_2_ adsorption processes, UiO-66@PAN10 and ZIF-8@PAN10 beads exhibit average desorption rates of 0.0160 and 0.0092 mmol g^–1^ min^–1^, respectively. When normalized with respect to the MOF loading only, the CO_2_ desorption rates for UiO-66@PAN10 (0.0178 mmol g^–1^ min^–1^) and ZIF-8@PAN10 (0.0102 mmol g^–1^ min^–1^) are also greater than those of pristine UiO-66 (0.0168 mmol g^–1^ min^–1^) and ZIF-8 powders (0.0098 mmol g^–1^ min^–1^). The improved CO_2_ adsorption and desorption kinetics observed for the MOFs integrated into the PAN matrix, compared to those of the pristine MOF powders, can be attributed to the dispersion of individual MOF particles within the PAN polymer matrix and the well-interconnected network of micron-sized channels, which provides easy access to the MOF adsorption sites.

The cyclability of the structured adsorbents was examined next to evaluate their suitability for practical cyclic adsorption-based CO_2_ capture applications. This was performed through VSA, where CO_2_ adsorption was recorded at 25 °C and 1 bar, followed by adsorbent regeneration through pressure reduction between cycles without any intermediate heat treatment. The adsorption and desorption isotherms of UiO-66@PAN10 (Figure S9b) and ZIF-8@PAN10 beads (Figure S9d) overlapped, indicating that CO_2_ could be readily desorbed from the structured composite beads by reducing the pressure without requiring external heating. To assess the performance of the structured composite beads over multiple cycles, the CO_2_ adsorption–desorption process was repeated for 10 VSA cycles at 25 °C, with pressure variations between 1 bar and vacuum. As shown in Figure 5c,d, the initial CO_2_ uptake capacity of the structured composite beads is retained after 10 cycles suggesting that the prepared structured composite beads hold promise for application in vacuum/pressure swing adsorption (V/PSA)-based CO_2_ capture without the need for thermal regeneration, while also providing ease of handling and processing compared with MOF powders.

For practical postcombustion CO_2_ capture applications, it is crucial to consider the presence of water vapor in the mixture, e.g., at a concentration of 5–10 vol % in flue gases.^9^ Therefore, MOFs should exhibit moisture stability and low moisture affinity because moisture can decrease their performance through competitive adsorption or destabilization of their crystalline structure from hydrolysis of the metal–ligand bonds. While both UiO-66 and ZIF-8 MOFs are water-stable, with ZIF-8 exhibiting also hydrophobic properties, whereas UiO-66 is hydrophilic. Therefore, to evaluate the water affinity of UiO-66@PAN10 relative to the UiO-66 powder, water adsorption isotherms were collected at 25 °C and up to 40% relative humidity (RH). As shown in Figure S10, the UiO-66@PAN10 beads exhibit enhanced hydrophobicity compared to UiO-66 powder, resulting in up to 31% reduction in water uptake capacity. This improvement is attributed to the existence of the protective PAN polymer matrix having higher hydrophobicity compared to the original UiO-66 powder, making UiO-66@PAN10 beads preferable to pure UiO-66 powders for CO_2_ capture from humidity-containing mixtures. This behavior aligns with the results of the TGA analysis (Figure S3), where a lower amount of weight loss associated with adsorbed water is observed in UiO-66@PAN10 than in UiO-66 powder. The enhancement of hydrophobicity is an added advantage, as it limits competitive water adsorption. A similar moisture protection strategy using a hydrophobic, but highly porous polymer can be applied to other moisture-sensitive adsorbents for CO_2_ capture from humidity-containing mixtures. This addresses the anticipated competitive adsorption between CO_2_ and H_2_O, as well as the destabilization of water-sensitive adsorbents due to exposure to water vapor.

### 3.3 Dynamic Breakthrough Studies

Assessing the dynamic CO_2_/N_2_ separation performance of adsorbents under continuous flow of a gas mixture is essential for the realistic evaluation of their potential for practical CO_2_ capture applications. The dynamic CO_2_ adsorption performance of the structured composite beads was evaluated through breakthrough experiments using a feed gas with a CO_2_/N_2_ volume ratio of 15/85, which is representative of coal power plant flue gas, at 25 °C, a total pressure of 1.25 bar, and a flow rate of 30 mL min^–1^, with helium as the carrier gas. UiO-66@PAN10 beads were selected for this study due to their high CO_2_ uptake capacity and ideal CO_2_/N_2_ selectivity among the structured composite beads in this work. Figure 5e shows the CO_2_ and N_2_ breakthrough curves for UiO-66@PAN10, while the CO_2_/N_2_ breakthrough curves for glass beads are presented in Figure S11a. As shown in Figure 5e, N_2_ is detected at the column outlet first, requiring a breakthrough time, defined as the time at which *C/C*_*0*_ = 0.05, of 101.9 s g^–1^, whereas the observed breakthrough time of CO_2_ is 186.9 s g^–1^, indicating that UiO-66@PAN10 preferentially adsorbs CO_2_ over N_2_ under dynamic flow conditions. The CO_2_/N_2_ selectivity is further quantified by calculating the dynamic CO_2_ and N_2_ adsorption capacities from the integration of the breakthrough curves from the initial time until pseudo equilibrium is reached, defined as the time at which C/C_0_ = 0.95, using eq 3. It was found that UiO-66@PAN10 has a dynamic CO_2_ and N_2_ adsorption capacity of 0.3 mmol g^–1^ and 0.1 mmol g^–1^, respectively, resulting in a dynamic CO_2_/N_2_ selectivity of 17, as calculated from eq 2. A similar selective CO_2_ adsorption over N_2_ was observed with a gas mixture having a CO_2_/N_2_ volume ratio of 25.5/74.5, typical of steel power plant flue gas, as shown in Figure S11b.

To address the demands of practical industrial applications, the dynamic cyclability and stability of the structured composite beads were evaluated by performing 10 consecutive cycles of breakthrough testing for the gas mixture with a CO_2_/N_2_ volume ratio of 15/85. Helium purging without external heating was used to regenerate the adsorbent bed after each cycle. As shown in Figure 5f, the CO_2_ and N_2_ breakthrough curves of the UiO-66@PAN10 for the 10 adsorption cycles overlapped, indicating that the structured composite bead maintained its CO_2_ uptake over repeated adsorption/desorption breakthrough tests and was successfully regenerated using helium purging without external heating. This confirms the dynamic cyclability of the developed structured composite beads under conditions that simulate a practical CO_2_ capture process. Additional evidence of structural stability was obtained through XRD, FTIR, and SEM analyses on the used structured adsorbents after 10 breakthrough cycles (Figure S12), demonstrating that their physical and chemical characteristics remain similar to those of fresh samples. Furthermore, N_2_ adsorption–desorption measurements at 77 K conducted after the cyclic studies (Figure S13) indicate that the porosity characteristics of UiO-66@PAN10 beads were maintained.

## 4 Conclusions

In this work, millimeter-sized hierarchically porous structured adsorbents with ultrahigh MOF loading (∼90 wt %) were developed for industrial CO_2_ capture application. A facile and versatile method was employed for integrating UiO-66 and ZIF-8, scalable microcrystalline MOFs with high moisture and thermal stability, with a PAN polymer matrix. CO_2_ and N_2_ adsorption measurements indicated that the porosity and CO_2_ uptake capacity of the parent MOF crystals were preserved, and the uniform dispersion of MOF particles on the macroporous PAN matrix facilitated easy access and exposure of their active sites to CO_2_ molecules. A specific surface area of 1130 m^2^ g^–1^ and CO_2_ adsorption capacity of 2.0 mmol g^–1^ at 1 bar and 25 °C were obtained for the structured UiO-66. A dynamic CO_2_/N_2_ selectivity of 17 for CO_2_/N_2_ gas mixture with 15/85 volume ratio at 25 °C was also obtained, indicating a CO_2_ selective adsorption potential. Furthermore, the structured composite beads exhibited excellent stability and cyclability across multiple static and dynamic breakthrough adsorption/desorption cycles without thermal regeneration, making them promising candidates for CO_2_ capture using vacuum/pressure swing adsorption (V/PSA) processes. This research contributes to the development of a scalable fabrication method for structured MOFs using commercially available polymers and materials. This approach not only preserves the structural integrity, porosity, and CO_2_ capture performance of pristine MOF crystals but also holds promise for scalability with a variety of other MOFs. This enables the processing of materials at a scale and paves the way for diverse practical applications beyond carbon capture, including water treatment and resource recovery. In addition to the proposed scalable structuring method, accelerating the deployment of MOFs for industrial applications requires optimizing the synthesis conditions of the microcrystalline MOFs to develop cost-effective synthesis methods with high yields.

## Supporting Information Available

Additional physicochemical characterization and CO_2_ adsorption performance testing data; Comparison of the surface area and pore volume of UiO-66 and ZIF-8 MOF powders with similar reported MOFs; Comparison of CO_2_ adsorption capacity of the structured adsorbents with reported structured MOFs (PDF)
