# Supplementary material for: Hierarchically Porous Structured Adsorbents with Ultrahigh Metal–Organic Framework Loading for CO2 Capture
Source: ACS Appl Mater Interfaces. 2024 Sep 16;16(38):50785–99. doi: 10.1021/acsami.4c10730 (PMC11440468; doi:10.1021/acsami.4c10730)
Supplement: Supplementary file 1 — am4c10730_si_001.pdf [file am4c10730_si_001.pdf]

Supporting information

Hierarchically-Porous Structured Adsorbents with Ultrahigh Metal-Organic Framework Loading for CO_2 Capture

Solomon K. Gebremariam[a,b], *Anish Mathai Varghese*[a,b], *Sebastian Ehrling*[c], *Yasser Al Wahedi*[d], *Ahmed AlHajaj*[a,e], *Ludovic F. Dumée*[a,f,*], *Georgios N. Karanikolos*[g,h*]

[a]Department of Chemical and Petroleum Engineering, Khalifa University, P.O. Box 127788, Abu Dhabi, United Arab Emirates

[b]Center for Catalysis and Separation (CeCaS), Khalifa University, P.O. Box 127788, Abu Dhabi, United Arab Emirates

[c]3P Instruments GmbH & Co. KG, Bitterfelder Str. 1-5, 04129 Leipzig, Germany

[d]Abu Dhabi Maritime Academy, P.O. Box 54477, Abu Dhabi, United Arab Emirates

[e]Research and Innovation Center on CO_2 and H_2 (RICH), Khalifa University, P.O. Box 127788, Abu Dhabi, United Arab Emirates

[f]Research and Innovation Center on 2D nanomaterials (RIC-2D), Khalifa University, Arzanah precinct, Sas Al Nakhl, P.O. Box 127788, Abu Dhabi, United Arab Emirates

[g]Department of Chemical Engineering, University of Patras, 26504 Patras, Greece

[h]Institute of Chemical Engineering Sciences, Foundation for Research and Technology-Hellas (FORTH/ICE-HT), 26504 Patras, Greece

[*]Corresponding authors Emails: ludovic.dumee@ku.ac.ae; karanikolos@chemeng.upatras.gr

XRD and FTIR analysis of structured ZIF-8 and UiO-66

The effect of the structuring process on the crystalline nature of the UiO-66 and ZIF-8 particles was investigated by XRD analysis of the adsorbents before and after structuring, as shown in Figure 2 a and b. The parent UiO-66 crystals display the characteristic XRD peaks corresponding to the UiO-66 crystal planes at 2θ values of 7.9 °, 9 °, 12.6 °, 17.6 °, 22.7 °, 26.3°, and 31.3 °,[1-2] and the parent ZIF-8 crystals at 2θ values of 7.8 °, 10.9 °, 13.3 °, 17 °, and 18.6 °.[3] The peaks of UiO-66@PAN and ZIF-8@PAN beads are in agreement to those of the UiO-66 and ZIF-8 powders, respectively, confirming preservation of the MOFs crystalline structure after integration into the PAN matrix.

The FTIR spectra of UiO-66 and ZIF-8 before and after structuring were collected (Figure 2 c and d) to evaluate the impact of structuring on the chemical characteristics of the adsorbents. For the UiO-66 powder, the bands at 1585 cm^{-1} and 1510 cm^{-1} correspond to the vibrations of the O-C=O and C=C bonds of the MOF ligand, respectively.[4-5] The band at 750 cm^{-1} is attributed to the stretching of the Zr-O-C bond, further confirming the formation of UiO-66 through the coordination of the oxygen atoms of the organic ligand with the zirconium cluster.[4, 6] For the ZIF-8 powder, the band at 1583 cm^{-1} is attributed to the C = N stretching mode,[7] whereas the bands in the range of 1350–1500 cm^{-1} correspond to imidazole ring stretching.[8] The bands in the range of 900–1350 cm^{-1} and at ∼760 cm^{-1} correspond to the in-plane[9] and out-of-plane[7] bending of the imidazole ring, respectively, while the band at 420 cm^{-1} corresponds to the Zn-N stretching mode,[8] further confirming the coordination of the zinc cations with the nitrogen atom of the ligand. The FTIR spectra of UiO-66@PAN and ZIF-8@PAN beads are analogous to those of UiO-66 and ZIF-8 powders, respectively, and the intensity of the bands increases with increasing loading of the MOF powders, indicating that the chemical characteristics of the individual UiO-66 and ZIF-8 particles are preserved in the MOF@PAN beads. All structured composites exhibit a band at 2240 cm^{-1}, corresponding to

the -C≡N stretching of PAN, [10] indicating the presence of PAN in the MOF@PAN beads. Notably, no new bands appeared in the FTIR spectra of the MOF@PAN beads, suggesting that physical interactions between the MOFs and PAN matrix play key roles in the formation of the structured adsorbents.

Comparison of the surface area and pore volume of ZIF-8 and UiO-66 with similar reported MOFs

The surface area and pore volume of the pure MOFs in this work (Table S1) are compared to those of similar MOFs reported in the literature. For UiO-66, the surface area in the present work is 1277 m^2 g^{-1}, which is comparable to or slightly lower than the values reported in other studies, such as 1367 m^2 g^{-1} [8], 1375 m^2 g^{-1} [1], 1434 m^2 g^{-1} [9] and 1580 m^2 g^{-1} [10]. On the other hand, lower surface areas have been reported, such as 241 m^2 g^{-1} [8], 838 m^2 g^{-1} [11], 1080 m^2 g^{-1} [12], 1105 m^2 g^{-1} [13] and 1110 m^2 g^{-1} [14]. The pore volume of UiO-66 in this work is 0.9 cm^3 g^{-1}, which is higher than values observed in other studies (0.245 cm^3 g^{-1} [11], 0.55 cm^3 g^{-1} [13], 0.56 cm^3 g^{-1} [8], 0.58 cm^3 g^{-1} [12], and 0.654 cm^3 g^{-1} [9]), while higher values (1.27 cm^3 g^{-1}) have also been reported [1]. The variations in surface area and pore volume among UiO-66 samples can be attributed to differences in synthetic methods, washing steps, and activation treatments [15]. For example, the traditional solvothermal synthesis method, which involves heating a mixture of zirconium chloride and terephthalic acid in DMF within a sealed vessel in an oven, is widely used and often yields surface areas ranging from 838 to 1110 m^2 g^{-1} [11-14]. High surface areas, such as 1434 m^2 g^{-1} [9], were achieved using chloroform activation, which effectively removes residual DMF and organic ligands, thereby enhancing surface area and N_2 adsorption capacity. The use of modulators, such as formic acid, has also significantly increased surface area, from 241 m^2 g^{-1} without formic acid to 1367 m^2 g^{-1} with formic acid [8]. Formic acid

accelerates crystal formation and improves crystallinity, leading to higher surface areas. Similarly, the addition of small amounts of HCl to the reaction mixture enhances the solubility of zirconium chloride in DMF [10], which leads to faster crystallization and potentially larger surface areas [1, 10]. For instance, a surface area of 1580 $m^2 g^{-1}$ using HCl additive was reported [10], where defect formation was noted as a reason for the higher surface area. Additionally, using HCl as additive and replacing zirconium chloride with $ZrOCl_2{\cdot}8H_2O$ has been associated with the formation of nano-sized UiO-66 particles with high surface areas (1375 $m^2 g^{-1}$) and pore volumes (1.27 $cm^3 g^{-1}$) [1]. In the present work, the synthesis of UiO-66 under reflux conditions with a small amount of HCl as a modulator likely contributed to the observed relatively high surface area (1277 $m^2 g^{-1}$) and pore volume (0.9 $cm^3 g^{-1}$). These results align with the trend that modulator additives can enhance the textural properties of MOFs. Furthermore, the high nitrogen uptake at high P/P_0 values and the associated high pore volume can also be attributed to the interparticular mesoporosity formed among the nanosized UiO-66 particles, as observed in the literature [1]. These observations highlight the important role of synthesis conditions, additives, and activation methods in tailoring the properties of UiO-66, including their N_2 uptake capacity.

Similarly, the surface area and pore volume of ZIF-8 prepared in the present work (1672 $m^2 g^{-1}$ and 0.72 $cm^3 g^{-1}$), using 2-methylimidazole ligand and $Zn(NO_3)_2{\cdot}4H_2O$ in methanol at room temperature, are comparable to or slightly higher than those reported for ZIF-8 prepared using analogous procedures, such as surface area of 1368 $m^2 g^{-1}$ [4], surface area of 1696 $m^2 g^{-1}$ with a micropore volume of 0.636 $cm^3 g^{-1}$ [16], and surface area of 1703 $m^2 g^{-1}$ with pore volume of 0.64 $cm^3 g^{-1}$ [17]. ZIF-8 prepared using a similar procedure but with the addition of n-butylamine as a modulator has been reported to have a surface area of 1617 $m^2 g^{-1}$ [18]. Additionally, surface areas and pore volumes for ZIF-8 prepared via various synthesis routes have been compared and reported [19], including solvothermal in methanol at room temperature

(1549 m^2 g^{-1} and 0.59 cm^3 g^{-1}), solvothermal in DMF at 140 °C (1370 m^2 g^{-1} and 0.51 cm^3 g^{-1}), microwave-assisted (1250 m^2 g^{-1} and 0.53 $cm^3 g^{-1}$), sonochemical (1249 m^2 g^{-1} and 0.71 cm^3 g^{-1}), and mechanochemical (1256 m^2 g^{-1} and 0.64 $cm^3 g^{-1}$). Other surface areas ranging from 962 to 1580 m^2 g^{-1} [19-20], micropore volumes from 0.32 to 0.7 cm^3 g^{-1} [20] and total pore volumes from 0.42 to 0.71 cm^3 g^{-1} [19] have also been reported for ZIF-8 prepared based on different synthetic methods, metal sources, reaction conditions, modulators, washing steps, and activation conditions. This variation underscores the importance of these parameters on the porosity and N_2 adsorption capacity of the MOF. Thus, the high surface area and pore volume of ZIF-8 obtained in the present work could be attributed to the solvothermal synthesis in methanol and effective washing steps, which provide improved textural properties and smaller particle sizes compared to methods such as solvothermal synthesis in DMF [19].

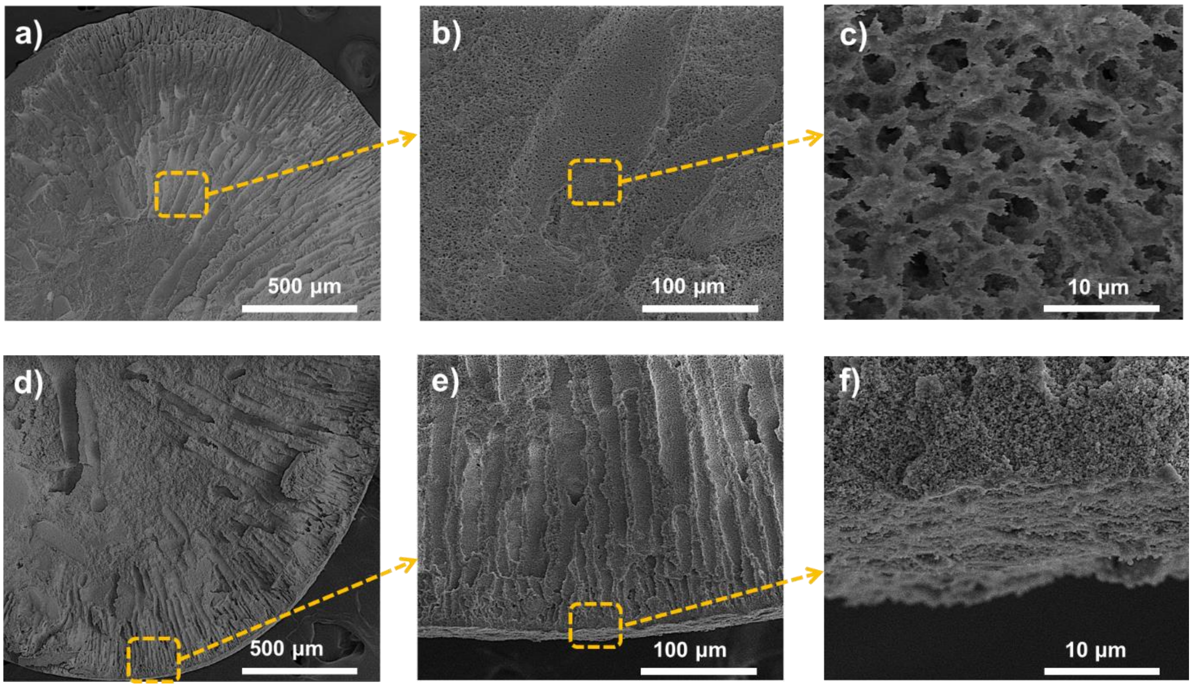

Figure S1. Cross-sectional SEM images of UiO-66@PAN10 beads: (a, d) Overall cross-sections at 500 μm magnification, illustrating the general bead structure. (b) Internal morphology at 100 μm magnification. (c) Detailed view of the internal structure at 10 μm magnification. (e) Cross-section near the surface at 100 μm magnification. (f) Cross-section of the surface skin at 10 μm magnification.

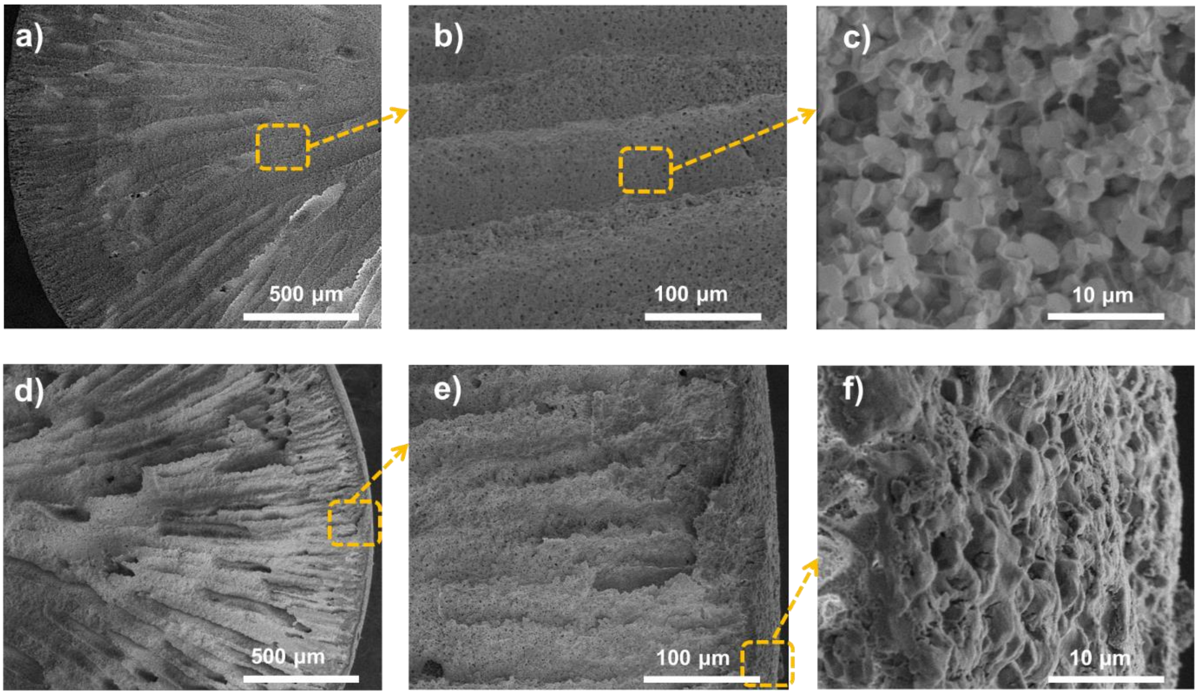

Figure S2. Cross-sectional SEM images of ZIF-8@PAN10 beads: (a, d) Overall cross-sections at 500 μm magnification, illustrating the general bead structure. (b) Internal morphology at 100 μm magnification. (c) Detailed view of the internal structure at 10 μm magnification. (e) Cross-section near the surface at 100 μm magnification. (f) Cross-section of the surface skin at 10 μm magnification.

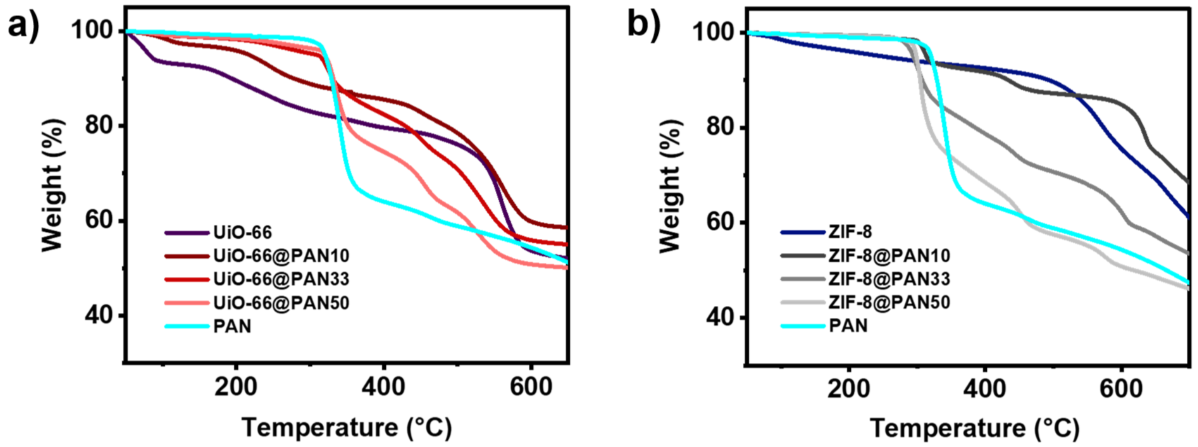

Figure S3. TGA curves of (a) UiO-66@PAN and (b) ZIF-8@PAN beads compared to their corresponding MOF powders.

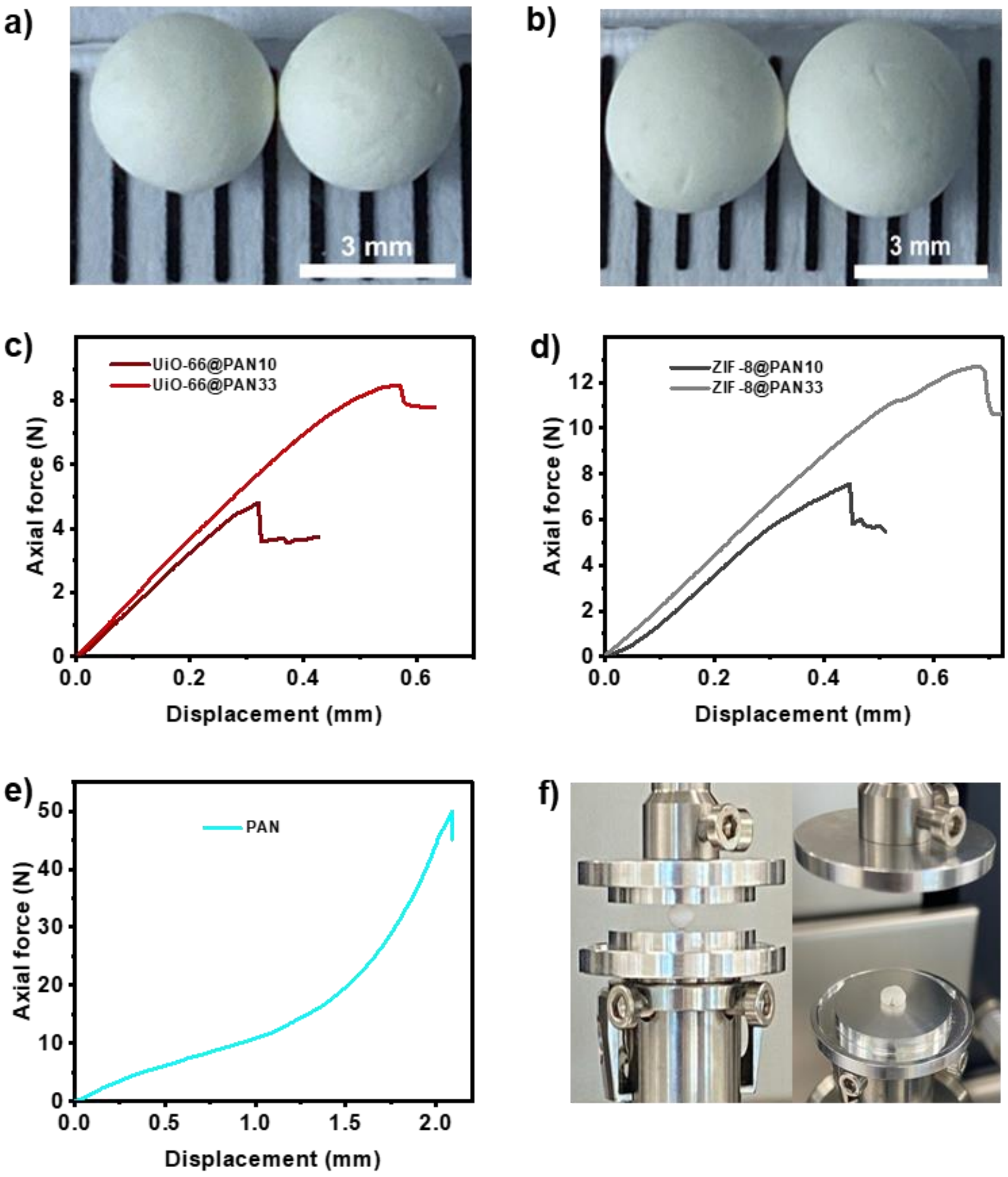

Figure S4. (a) UiO-66@PAN10 and (b) ZIF-8@PAN10 beads after exposure to 10 bar of N_2 for 16 h, showing no visual breakage, demonstrating their stability to sustain high pressure. Mechanical testing results showing representative axial force versus displacement curves for (c) UiO-66@PAN, (d) ZIF-8@PAN, and (e) neat PAN beads. (f) Photograph of the testing setup for crushing tests of structured composite beads, showing a single bead placed between 25 mm diameter parallel plates, both before and after the crushing test.

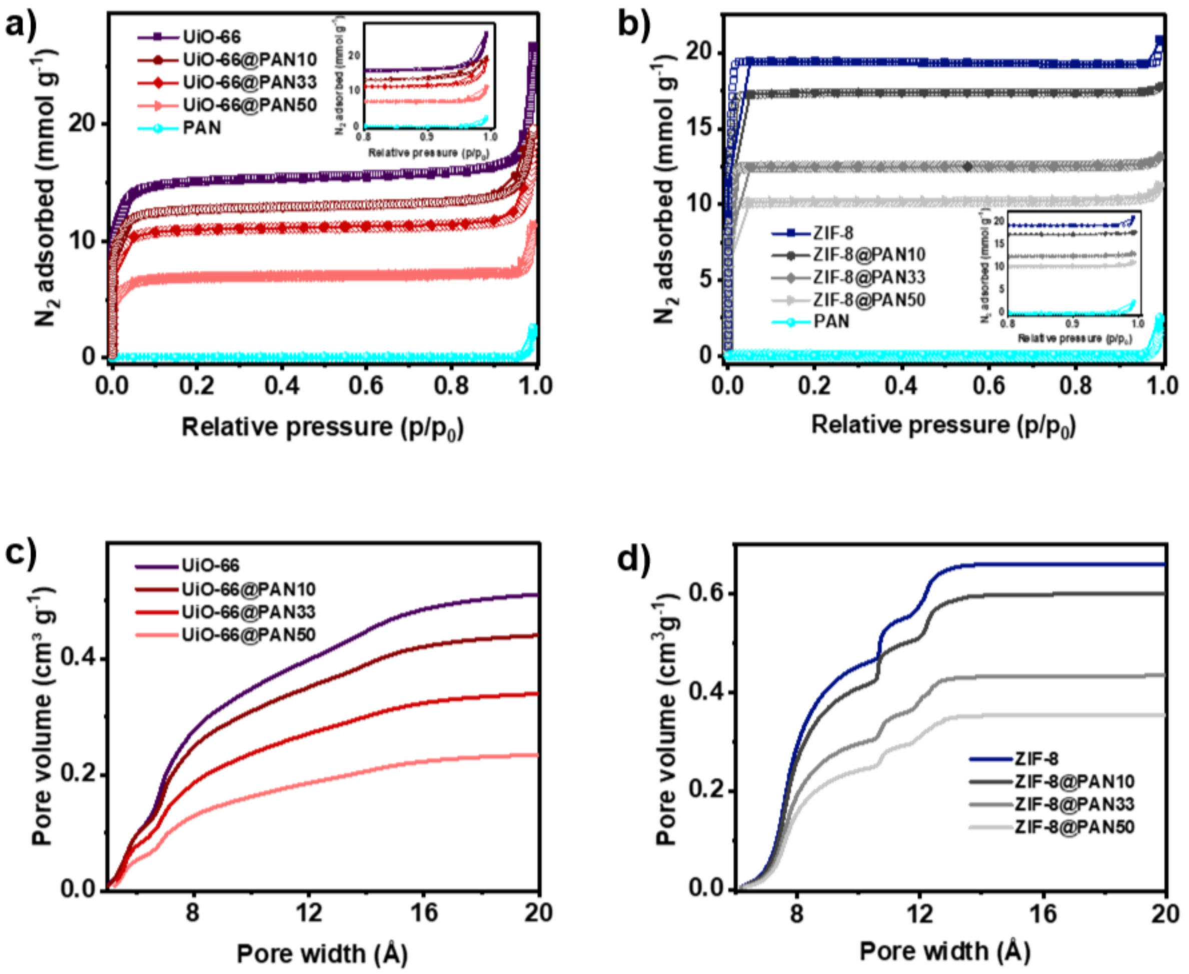

Figure S5. N_2 adsorption–desorption isotherms at 77 K for (a) UiO-66@PAN and (b) ZIF-8@PAN beads in comparison to their respective MOF powders. Insets: high-resolution N_2 adsorption–desorption isotherms at high relative pressure. Cumulative micropore size distributions for (c) UiO-66@PAN and (d) ZIF-8@PAN beads in comparison to their respective MOF powders. Data presented per unit of total mass of the MOF@PAN beads.

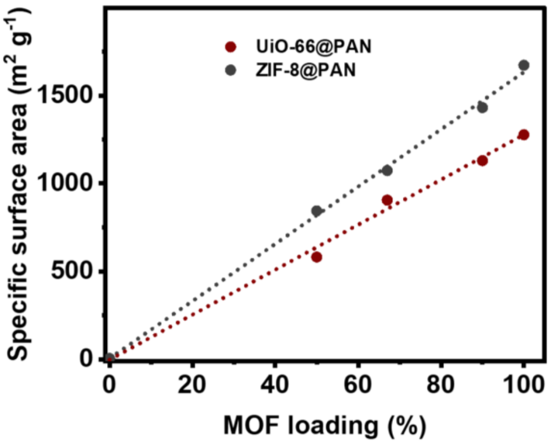

Figure S6. BET surface area vs. MOF loading for the structured adsorbents. The expected surface area, calculated based on the mixing rule, is represented by the dashed line.

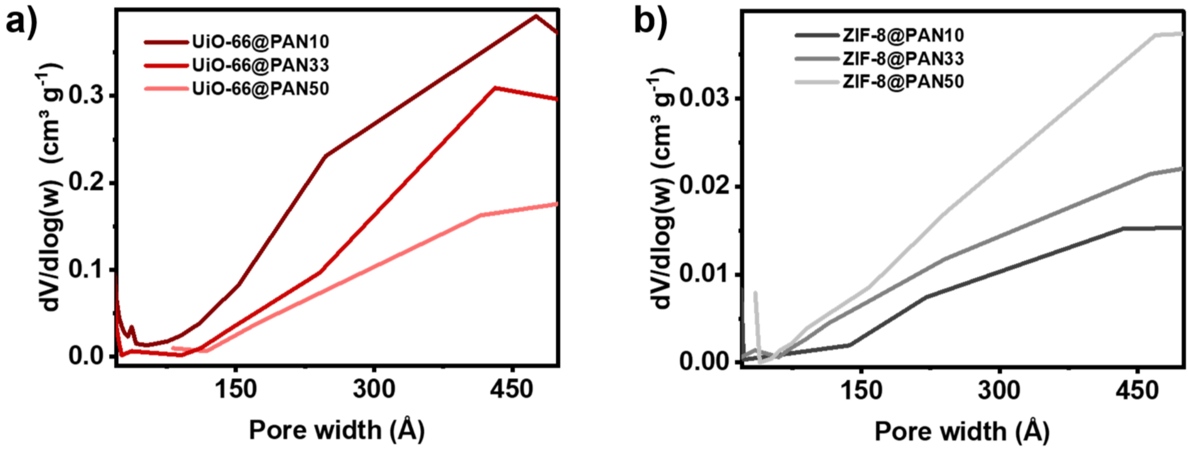

Figure S7. Mesopore size distribution derived by BJH desorption for (a) UiO-66@PAN and (b) ZIF-8@PAN beads.

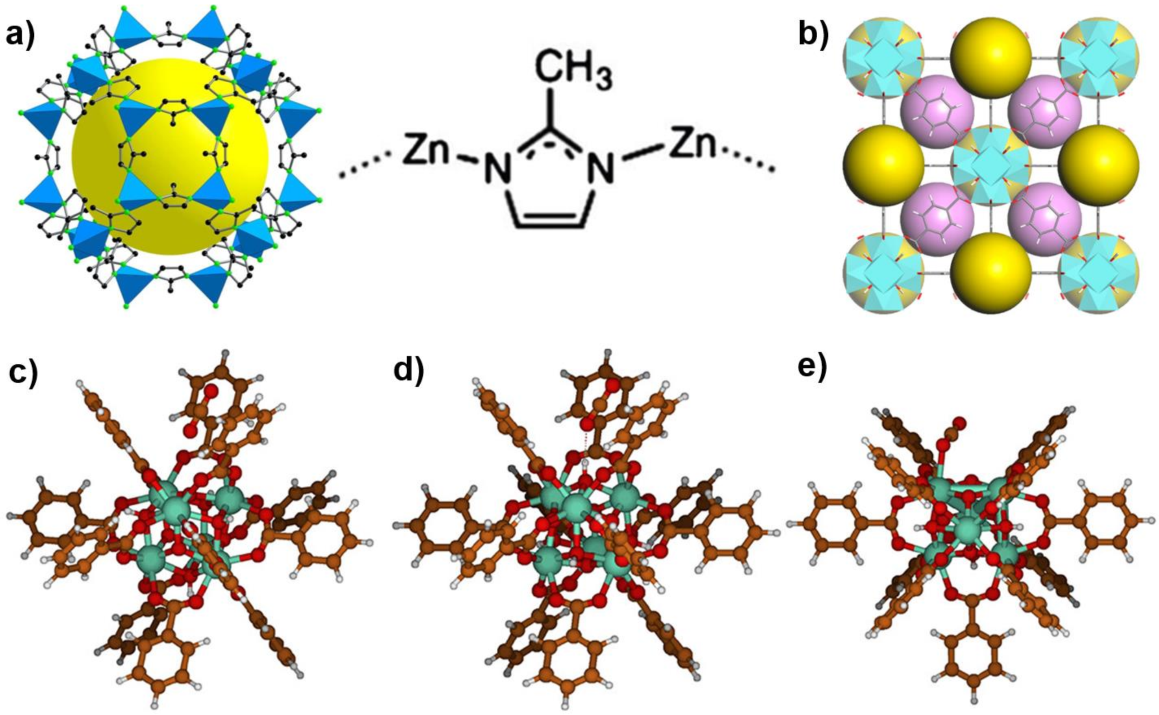

Figure S8. (a) Crystal structure of ZIF-8, featuring Zn metal centers connected by 2-methylimidazole linkers. Color code: Zn (polyhedral), N (green), C (black), with H omitted for simplicity. The large yellow sphere indicates a free space in the framework. Reprinted with permission from [26]. Copyright (2019) Elsevier. (b) Crystal structure of UiO-66, showing Zr atoms coordinated with terephthalic acid linkers. The large yellow and pink spheres represent the octahedral and tetrahedral cavities in the MOF, respectively. Color code: Zr (cyan), C (gray), O (red), and H (white). Reprinted with permission from [27]. Copyright (2013) American Chemical Society. CO_2 adsorption mechanisms in UiO-66: (c) dispersion interactions within the confined pores, (d) hydrogen bonding with -OH groups, and (e) binding to open zirconium sites formed due to defects or missing linkers. Color code: Zr (cyan), O (red), H (white), C (brown). Reprinted with permission from [28]. Copyright (2019) American Chemical Society.

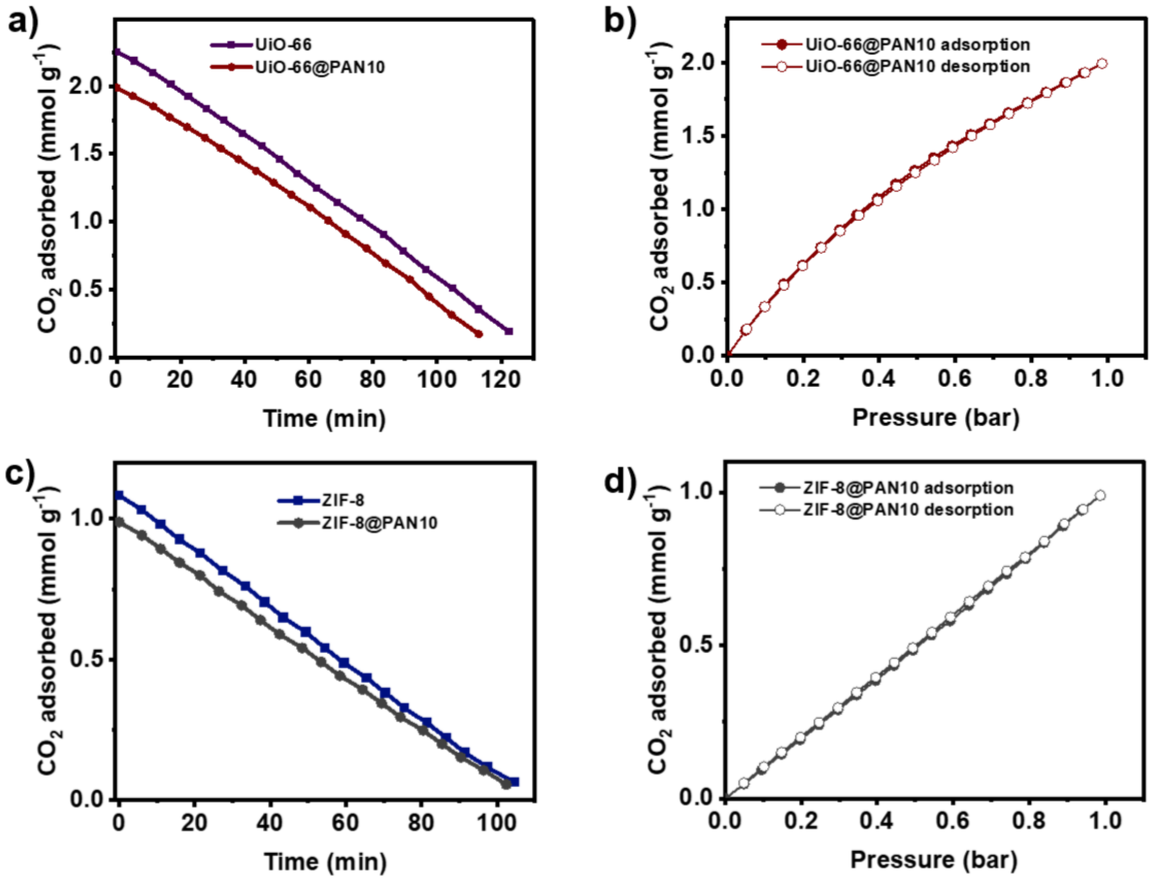

Figure S9. CO_2 desorption kinetics for (a) UiO-66@PAN10 and Vacuum swing adsorption (VSA)-based CO_2 adsorption/desorption isotherms at 1 bar and 25 °C for (b) UiO-66@PAN10. CO_2 desorption kinetics for (c) ZIF-8@PAN10 and VSA-based CO_2 adsorption/desorption isotherms at 1 bar and 25 °C for (d) ZIF-8@PAN10.

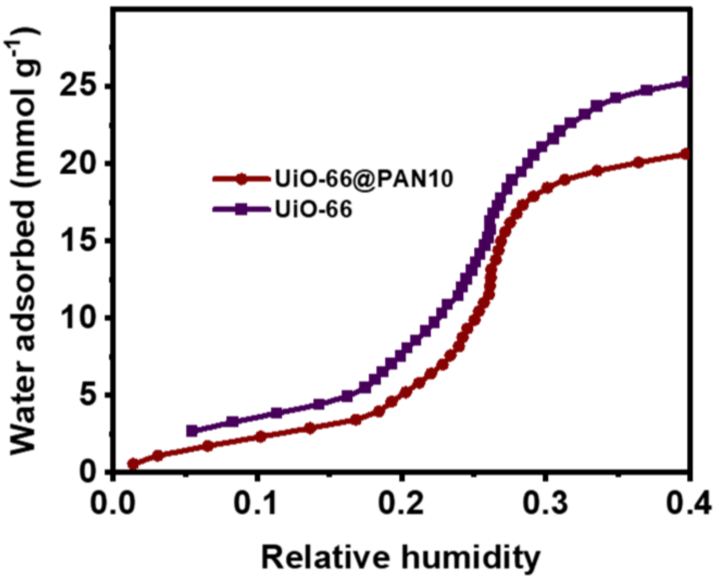

Figure S10. Water adsorption isotherms for UiO-66@PAN10 and UiO-66 powder at 25 °C and 1 bar.

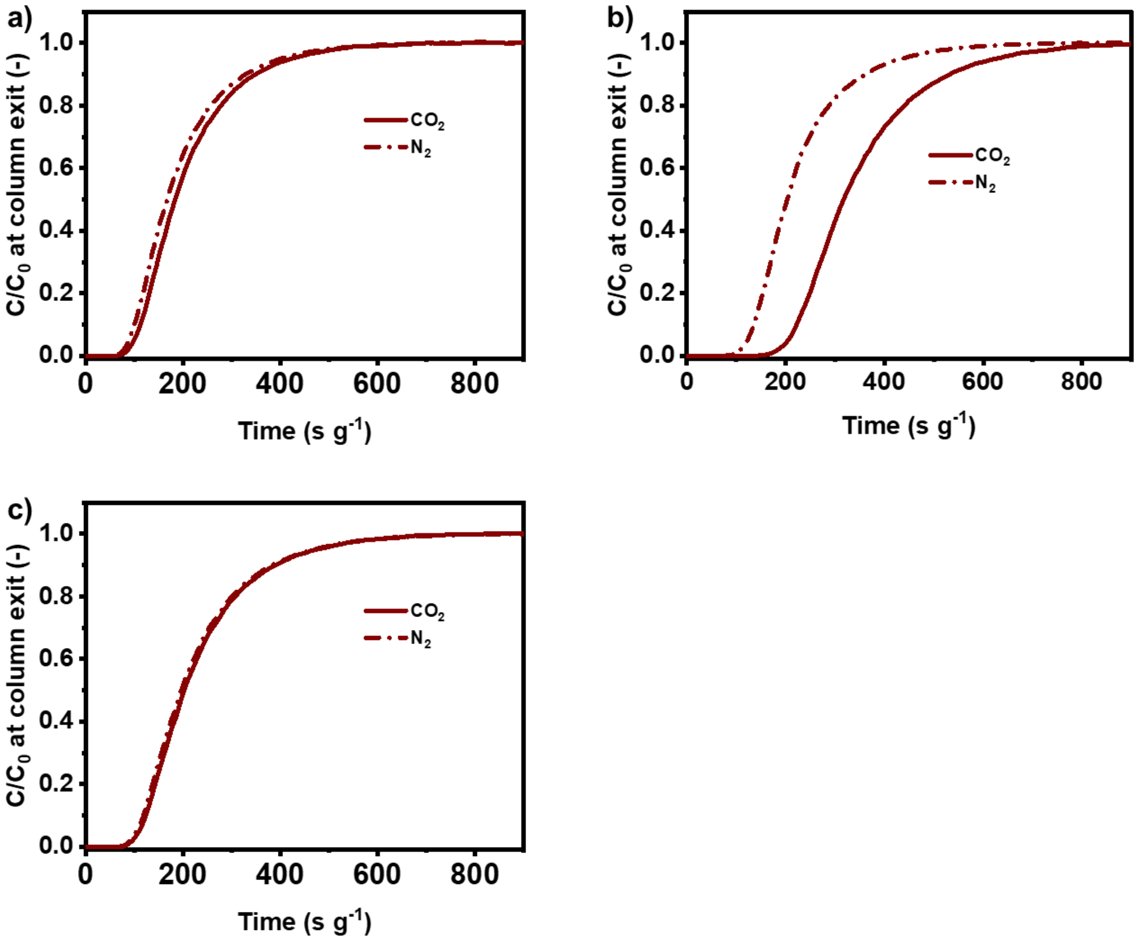

Figure S11. CO_2 and N_2 breakthrough curves at 25 °C, a flow rate of 30 ml min^{-1}, and a total pressure of 1.25 bar with helium as the carrier gas for: (a) glass beads using a gas mixture with a CO_2/N_2 volume ratio of 15/85; (b) UiO-66@PAN10 using a gas mixture with a CO_2/N_2 volume ratio of 25.5/74.5; and (c) glass beads using a gas mixture with a CO_2/N_2 volume ratio of 25.5/74.5.

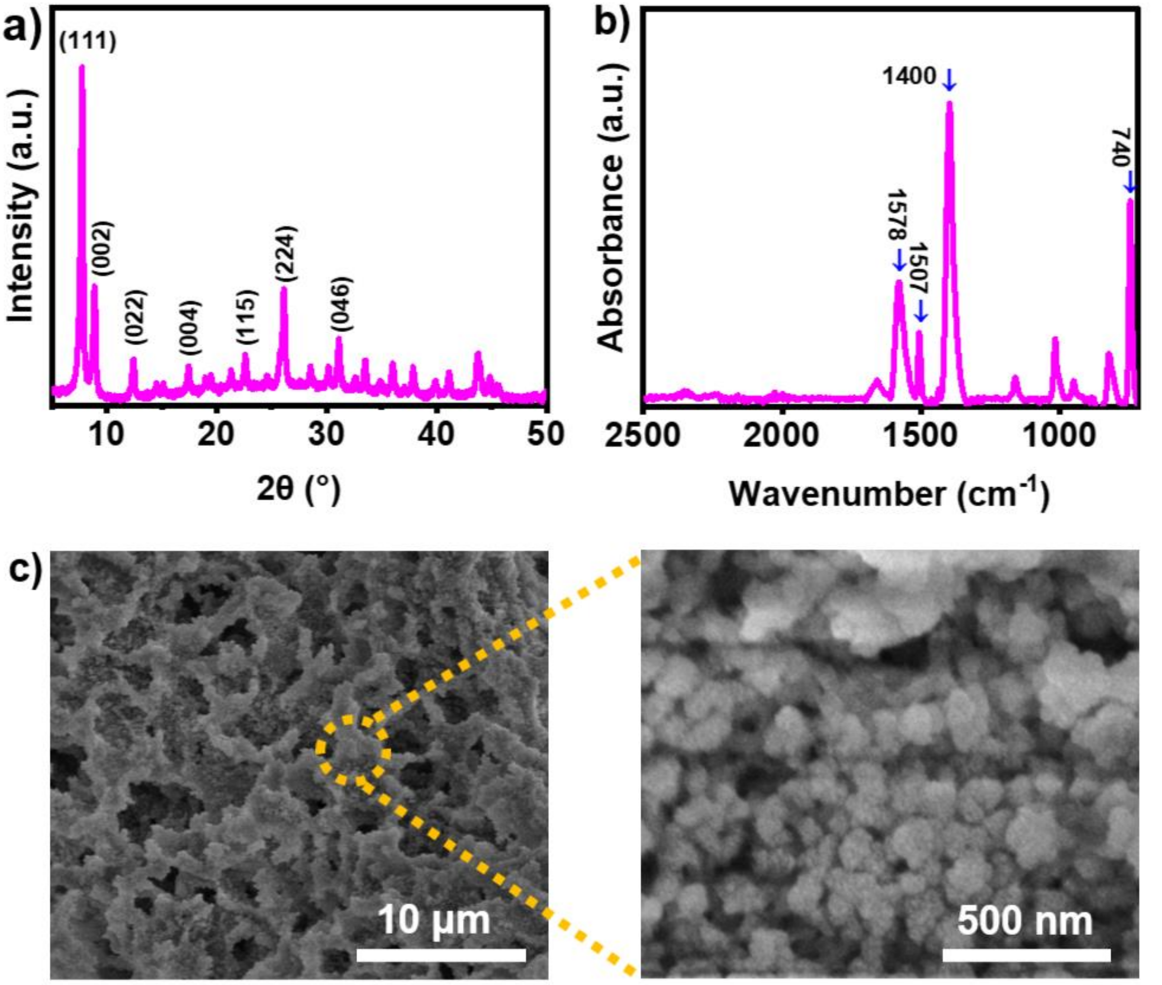

Figure S12. (a) XRD pattern, (b) FTIR spectrum, and (c) SEM images for UiO-66@PAN10 after 10 breakthrough cycles.

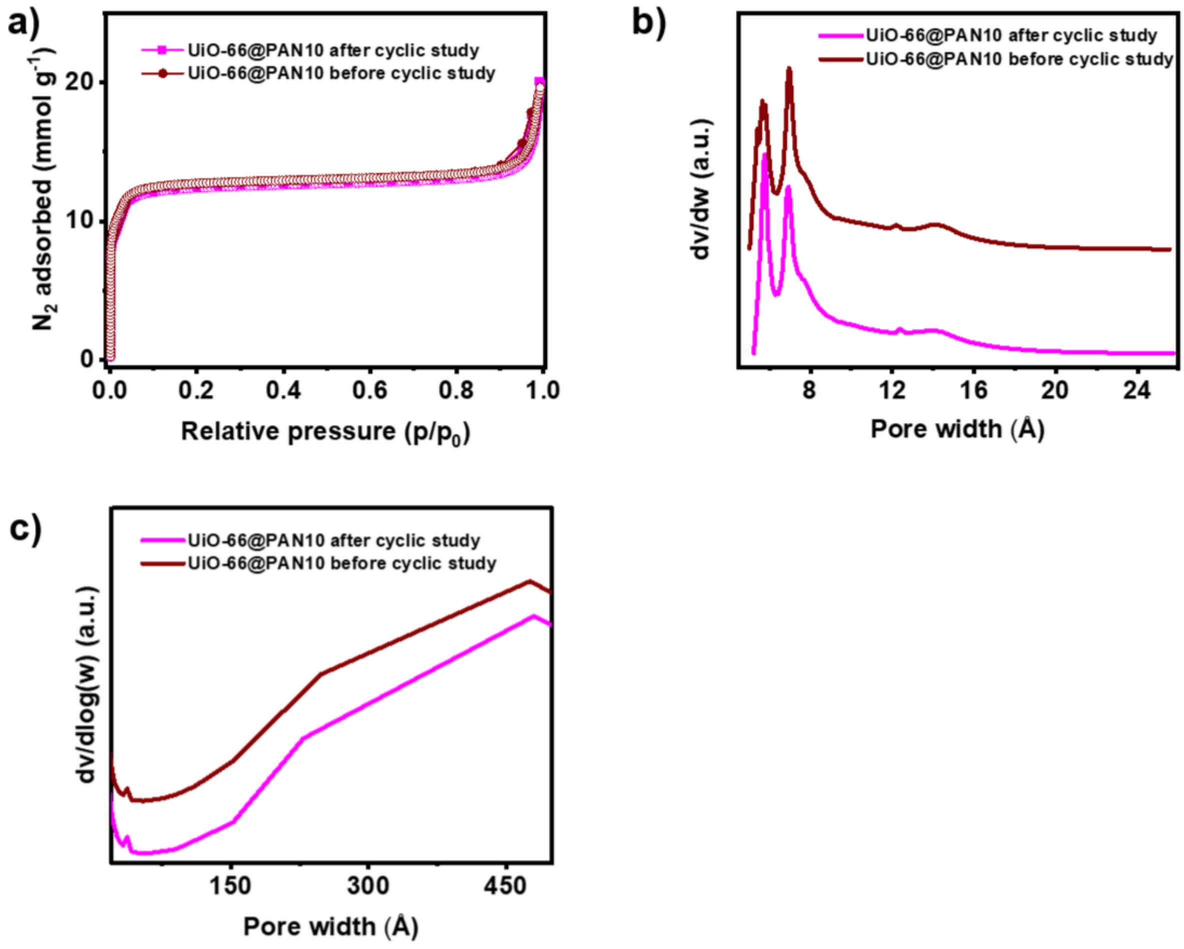

Figure S13. (a) N_2 adsorption-desorption isotherm, (b) micropore size distribution, and (c) mesopore size distribution of UiO-66@PAN10 before and after 10 breakthrough cycles.

Table S1. Textural properties of UiO-66@PAN and ZIF-PAN beads, in comparison to their corresponding MOF powders and pure PAN beads. Data presented per unit of total mass of the MOF@PAN beads.

Adsorbent	Specific surface area [m^2 g^{-1}]	Total pore volume [cm^3 g^{-1}]	t-plot micropore volume [cm^3 g^{-1}]	Average micropore diameter [Å]
**PAN**	4.62	0.08	-	-
**UiO-66@PAN50**	581	0.38	0.21	7.7
**UiO-66@PAN33**	905.13	0.66	0.34	7.7
**UiO-66@PAN10**	1130	0.69	0.40	7.7
**UiO-66 powder**	1277	0.9	0.45	7.8
**ZIF-8@PAN50**	844	0.38	0.35	8.3
**ZIF-8@PAN33**	1073	0.46	0.43	8.3
**ZIF-8@PAN10**	1431	0.62	0.60	8.3
**ZIF-8 powder**	1672	0.72	0.68	8.3

Table S2. Equilibrium CO_2 adsorption capacities of structured UiO-66 and ZIF-8 MOFs compared to reported structured composites, listed at 1 bar and 25 °C in ascending order, and at 0.15 bar and 25 °C. The data for 0.15 bar have been extracted from CO_2 adsorption isotherm plots.

Adsorbent	CO_2 uptake [mmol g^{-1}] at 1 bar	CO_2 uptake [mmol g^{-1}] at 0.15 bar	References
CSGO aerogels	0.26	0.06	[29]
PI/ZIF composite aerogels	0.4	0.11	[30]
MOF-177/PVB pellets	0.55	-	[31]
Chitosan/ZIF-8 composites beads	0.56	0.1	[32]
CTS-GO-5% aerogels	0.6	-	[33]
MCM-41/CHIT 50%	0.61	-	[34]
nanocellulose/ZIF-based foams	0.62	0.2	[35]
PIL-chitosan aerogels	0.7	-	[36]
PI/ZIF-8 aerogels	0.72	0.12	[37]
ZIF-67/CS2:1 cryogels	0.76	-	[38]
graphene/ZIF-8 aerogel	0.99	0.2	[39]
ZIF-8@PAN10 beads	1	0.16	This work
UTSA-16(Co)-cordierite monolith	1.1	0.65	[40]
HKUST-1@Torlon monolith	1.2	0.3	[41]
MIL-101(Cr)@GO composite beads	1.66	0.33	[42]
MOF-74(Ni)-cordierite monolith	1.7	1.2	[40]

HKUST-1@CS beads	1.98	0.4	[43]
SIFSIX-3-Cu@MFS	2.0	1.8	[44]
UiO-66@PAN10 beads	2.0	0.44	This work
MOF-74−polyHIPE composite monoliths	2.2	1.5	[45]
Mg-MOF-74/SA monolith composite	2.5	1.2	[46]

References

1. Wen, L.; Chen, X.; Chen, C.; Yang, R.; Gong, M.; Zhang, Y.; Fu, Q., Ice-templated porous polymer/UiO-66 monolith for Congo Red adsorptive removal. *Arab. J. Chem.* **2020,** *13* (6), 5669-5678.

2. Cavka, J. H.; Jakobsen, S.; Olsbye, U.; Guillou, N.; Lamberti, C.; Bordiga, S.; Lillerud, K. P., A new zirconium inorganic building brick forming metal organic frameworks with exceptional stability. *JACS* **2008,** *130* (42), 13850-13851.

3. Zhu, L.; Zong, L.; Wu, X.; Li, M.; Wang, H.; You, J.; Li, C., Shapeable fibrous aerogels of metal–organic-frameworks templated with nanocellulose for rapid and large-capacity adsorption. *ACS nano* **2018,** *12* (5), 4462-4468.

4. Vo, T. K.; Le, V. N.; Yoo, K. S.; Song, M.; Kim, D.; Kim, J., Facile synthesis of UiO-66 (Zr) using a microwave-assisted continuous tubular reactor and its application for toluene adsorption. *Cryst. Growth Des.* **2019,** *19* (9), 4949-4956.

5. Hu, P.; Liang, X.; Yaseen, M.; Sun, X.; Tong, Z.; Zhao, Z.; Zhao, Z., Preparation of highly-hydrophobic novel N-coordinated UiO-66 (Zr) with dopamine via fast mechano-

chemical method for (CHO-/Cl-)-VOCs competitive adsorption in humid environment. *Chem. Eng. J.* **2018,** *332*, 608-618.

6. Vo, T. K.; Nguyen, V. C.; Song, M.; Kim, D.; Yoo, K. S.; Park, B. J.; Kim, J., Microwave-assisted continuous-flow synthesis of mixed-ligand UiO-66 (Zr) frameworks and their application to toluene adsorption. *J. Ind. Eng. Chem.* **2020,** *86*, 178-185.

7. Abbasi, Z.; Shamsaei, E.; Leong, S. K.; Ladewig, B.; Zhang, X.; Wang, H., Effect of carbonization temperature on adsorption property of ZIF-8 derived nanoporous carbon for water treatment. *Micropor Mesopor Mat* **2016,** *236*, 28-37.

8. Chen, Y.; Tang, S., Solvothermal synthesis of porous hydrangea-like zeolitic imidazole framework-8 (ZIF-8) crystals. *J. Solid State Chem.* **2019,** *276*, 68-74.

9. Niknam Shahrak, M.; Ghahramaninezhad, M.; Eydifarash, M., Zeolitic imidazolate framework-8 for efficient adsorption and removal of Cr (VI) ions from aqueous solution. *ESPR* **2017,** *24*, 9624-9634.

10. Zhang, Y.; Guan, J.; Wang, X.; Yu, J.; Ding, B., Balsam-pear-skin-like porous polyacrylonitrile nanofibrous membranes grafted with polyethyleneimine for postcombustion CO2 capture. *ACS Appl. Mater. Interfaces* **2017,** *9* (46), 41087-41098.

11. Ren, J.; Musyoka, N. M.; Langmi, H. W.; Swartbooi, A.; North, B. C.; Mathe, M., A more efficient way to shape metal-organic framework (MOF) powder materials for hydrogen storage applications. *Int. J. Hydrogen Energy.* **2015,** *40* (13), 4617-4622.

12. Ragon, F.; Horcajada, P.; Chevreau, H.; Hwang, Y. K.; Lee, U.-H.; Miller, S. R.; Devic, T.; Chang, J.-S.; Serre, C., In situ energy-dispersive X-ray diffraction for the synthesis optimization and scale-up of the porous zirconium terephthalate UiO-66. *Inorg. Chem.* **2014,** *53* (5), 2491-2500.

13. Abid, H. R.; Tian, H.; Ang, H.-M.; Tade, M. O.; Buckley, C. E.; Wang, S., Nanosize Zr-metal organic framework (UiO-66) for hydrogen and carbon dioxide storage. *Chem. Eng. J.* **2012,** *187*, 415-420.

14. Katz, M. J.; Brown, Z. J.; Colón, Y. J.; Siu, P. W.; Scheidt, K. A.; Snurr, R. Q.; Hupp, J. T.; Farha, O. K., A facile synthesis of UiO-66, UiO-67 and their derivatives. *ChemComm* **2013,** *49* (82), 9449-9451.

15. Cao, Y.; Zhao, Y.; Lv, Z.; Song, F.; Zhong, Q., Preparation and enhanced CO2 adsorption capacity of UiO-66/graphene oxide composites. *J. Ind. Eng. Chem.* **2015,** *27*, 102-107.

16. Abdelmoaty, A. S.; El-Wakeel, S. T.; Fathy, N.; Hanna, A. A., High performance of UiO-66 metal–organic framework modified with melamine for uptaking of lead and cadmium from aqueous solutions. *Journal of Inorganic and Organometallic Polymers and Materials* **2022,** *32* (7), 2557-2567.

17. Cmarik, G. E.; Kim, M.; Cohen, S. M.; Walton, K. S., Tuning the adsorption properties of UiO-66 via ligand functionalization. *Langmuir* **2012,** *28* (44), 15606-15613.

18. Garibay, S. J.; Cohen, S. M., Isoreticular synthesis and modification of frameworks with the UiO-66 topology. *ChemComm* **2010,** *46* (41), 7700-7702.

19. Cox, C. S.; Slavich, E.; Macreadie, L. K.; McKemmish, L. K.; Lessio, M., Understanding the role of synthetic parameters in the defect engineering of UiO-66: a review and meta-analysis. *Chem. Mater.* **2023,** *35* (8), 3057-3072.

20. Sun, H.; Tang, B.; Wu, P., Hydrophilic hollow zeolitic imidazolate framework-8 modified ultrafiltration membranes with significantly enhanced water separation properties. *J. Membr. Sci.* **2018,** *551*, 283-293.

21. Demessence, A.; Boissière, C.; Grosso, D.; Horcajada, P.; Serre, C.; Férey, G.; Soler-Illia, G. J.; Sanchez, C., Adsorption properties in high optical quality nanoZIF-8 thin films with tunable thickness. *J. Mater. Chem.* **2010,** *20* (36), 7676-7681.

22. Erkartal, M.; Erkilic, U.; Tam, B.; Usta, H.; Yazaydin, O.; Hupp, J. T.; Farha, O. K.; Sen, U., From 2-methylimidazole to 1, 2, 3-triazole: a topological transformation of ZIF-8 and ZIF-67 by post-synthetic modification. *ChemComm* **2017,** *53* (12), 2028-2031.

23. Cravillon, J.; Nayuk, R.; Springer, S.; Feldhoff, A.; Huber, K.; Wiebcke, M., Controlling zeolitic imidazolate framework nano-and microcrystal formation: insight into crystal growth by time-resolved in situ static light scattering. *Chem. Mater.* **2011,** *23* (8), 2130-2141.

24. Lee, Y.-R.; Jang, M.-S.; Cho, H.-Y.; Kwon, H.-J.; Kim, S.; Ahn, W.-S., ZIF-8: A comparison of synthesis methods. *Chem. Eng. J.* **2015,** *271*, 276-280.

25. Jin, C.-X.; Shang, H.-B., Synthetic methods, properties and controlling roles of synthetic parameters of zeolite imidazole framework-8: A review. *J. Solid State Chem.* **2021,** *297*, 122040.

26. Liu, Y.; Cheng, H.; Cheng, M.; Liu, Z.; Huang, D.; Zhang, G.; Shao, B.; Liang, Q.; Luo, S.; Wu, T., The application of Zeolitic imidazolate frameworks (ZIFs) and their derivatives based materials for photocatalytic hydrogen evolution and pollutants treatment. *Chem. Eng. J.* **2021,** *417*, 127914.

27. Wu, H.; Chua, Y. S.; Krungleviciute, V.; Tyagi, M.; Chen, P.; Yildirim, T.; Zhou, W., Unusual and highly tunable missing-linker defects in zirconium metal–organic framework UiO-66 and their important effects on gas adsorption. *JACS* **2013,** *135* (28), 10525-10532.

28. Grissom, T. G.; Driscoll, D. M.; Troya, D.; Sapienza, N. S.; Usov, P. M.; Morris, A. J.; Morris, J. R., Molecular-level insight into CO2 adsorption on the zirconium-based metal–

organic framework, UiO-66: a combined spectroscopic and computational approach. *J. Phys. Chem. C* **2019,** *123* (22), 13731-13738.

29. Hsan, N.; Dutta, P.; Kumar, S.; Bera, R.; Das, N., Chitosan grafted graphene oxide aerogel: Synthesis, characterization and carbon dioxide capture study. *Int. J. Biol. Macromol.* **2019,** *125*, 300-306.

30. Wu, T.; Dong, J.; De France, K.; Zhang, P.; Zhao, X.; Zhang, Q., Porous carbon frameworks with high CO2 capture capacity derived from hierarchical polyimide/zeolitic imidazolate frameworks composite aerogels. *Chem. Eng. J.* **2020,** *395*, 124927.

31. Gaikwad, S.; Kim, Y.; Gaikwad, R.; Han, S., Enhanced CO2 capture capacity of amine-functionalized MOF-177 metal organic framework. *J. Environ. Chem. Eng.* **2021,** *9* (4), 105523.

32. Yao, J.; Chen, R.; Wang, K.; Wang, H., Direct synthesis of zeolitic imidazolate framework-8/chitosan composites in chitosan hydrogels. *Micropor Mesopor Mat* **2013,** *165*, 200-204.

33. Alhwaige, A. A.; Agag, T.; Ishida, H.; Qutubuddin, S., Biobased chitosan hybrid aerogels with superior adsorption: Role of graphene oxide in CO 2 capture. *RSC Adv.* **2013,** *3* (36), 16011-16020.

34. Oliveira, D. E.; Chagas, J. A.; De Lima, A. L.; Mota, C. J., CO2 capture over MCM-41 and SBA-15 mesoporous silicas impregnated with chitosan. *Ind. Eng. Chem. Res.* **2022,** *61* (29), 10522-10530.

35. Valencia, L.; Abdelhamid, H. N., Nanocellulose leaf-like zeolitic imidazolate framework (ZIF-L) foams for selective capture of carbon dioxide. *Carbohydr. Polym.* **2019,** *213*, 338-345.

36. Barrulas, R. V.; López-Iglesias, C.; Zanatta, M.; Casimiro, T.; Mármol, G.; Carrott, M. R.; García-González, C. A.; Corvo, M. C., The AEROPILs Generation: Novel Poly (Ionic Liquid)-Based Aerogels for CO2 Capture. *Int. J. Mol. Sci.* **2021,** *23* (1), 200.

37. Zhang, Z.; Zhang, J.; Dou, G.; Zeng, Q., Synthesis of PI/ZIF-8 aerogel with hierarchical porous structure for enhanced CO2 capture performance. *Chem. Phys. Lett.* **2022,** *801*, 139703.

38. Hammi, N.; Couzon, N.; Loiseau, T.; Volkringer, C.; El Kadib, A.; Royer, S.; Dhainaut, J., Hierarchically porous ZIF-67/chitosan beads with high surface area and strengthened mechanical properties: Application to CO2 storage. *Mater Today Sustain* **2023,** *22*, 100394.

39. Jiang, M.; Li, H.; Zhou, L.; Xing, R.; Zhang, J., Hierarchically porous graphene/ZIF-8 hybrid aerogel: preparation, CO2 uptake capacity, and mechanical property. *ACS Appl. Mater. Interfaces* **2018,** *10* (1), 827-834.

40. Rezaei, F.; Lawson, S.; Hosseini, H.; Thakkar, H.; Hajari, A.; Monjezi, S.; Rownaghi, A. A., MOF-74 and UTSA-16 film growth on monolithic structures and their CO2 adsorption performance. *Chem. Eng. J.* **2017,** *313*, 1346-1353.

41. Lawson, S.; Snarzyk, M.; Hanify, D.; Rownaghi, A. A.; Rezaei, F., Development of 3D-printed polymer-MOF monoliths for CO2 adsorption. *Ind. Eng. Chem. Res.* **2019,** *59* (15), 7151-7160.

42. Gebremariam, S. K.; Varghese, A. M.; Reddy, K. S. K.; AlWahedi, Y. F.; Dumée, L. F.; Karanikolos, G. N., Polymer-aided microstructuring of moisture-stable GO-hybridized MOFs for carbon dioxide capture. *Chem. Eng. J.* **2023,** *473*, 145286.

43. Hammi, N.; Bonneau, M.; El Kadib, A.; Kitagawa, S.; Loiseau, T.; Volkringer, C.; Royer, S.; Dhainaut, J., Enhanced Gas Adsorption in HKUST-1@ Chitosan Aerogels, Cryogels, and Xerogels: An Evaluation Study. *ACS Appl. Mater. Interfaces* **2023**.

44. Zhen, H.-G.; Mao, H.; Haq, I. U.; Li, S.-H.; Ahmad, A.; Zhao, Z.-P., In-situ SIFSIX-3-Cu growth into melamine formaldehyde sponge monolith for CO2 efficient capture. *Sep. Purif. Technol.* **2020,** *233*, 116042.

45. Vrtovec, N.; Jurjevec, S.; Zabukovec Logar, N. a.; Mazaj, M.; Kovačič, S., Metal oxide-derived MOF-74 polymer composites through pickering emulsion-templating: interfacial recrystallization, hierarchical architectures, and CO2 capture performances. *ACS Appl. Mater. Interfaces* **2023,** *15* (14), 18354-18361.

46. Peng, X.; Zhang, J.; Sun, J.; Liu, X.; Zhao, X.; Yu, S.; Yuan, Z.; Liu, S.; Yi, X., Hierarchically Porous Mg-MOF-74/Sodium Alginate Composite Aerogel for CO2 Capture. *ACS Appl. Nano Mater.* **2023,** *6* (18), 16694-16701.
